# Concerted action of ataxin-2 and PABPC1-bound mRNA poly(A) tail in the formation of stress granules

**DOI:** 10.1093/nar/gkae497

**Published:** 2024-06-13

**Authors:** Ryota Yamagishi, Hiroto Inagaki, Jun Suzuki, Nao Hosoda, Haruka Sugiyama, Kazunori Tomita, Takashi Hotta, Shin-ichi Hoshino

**Affiliations:** Department of Biological Chemistry, Graduate School of Pharmaceutical Sciences, Nagoya City University, Nagoya 467-8603, Japan; Department of Biological Chemistry, Graduate School of Pharmaceutical Sciences, Nagoya City University, Nagoya 467-8603, Japan; Department of Biological Chemistry, Graduate School of Pharmaceutical Sciences, Nagoya City University, Nagoya 467-8603, Japan; Department of Biological Chemistry, Graduate School of Pharmaceutical Sciences, Nagoya City University, Nagoya 467-8603, Japan; Department of Biological Chemistry, Graduate School of Pharmaceutical Sciences, Nagoya City University, Nagoya 467-8603, Japan; Department of Biological Chemistry, Graduate School of Pharmaceutical Sciences, Nagoya City University, Nagoya 467-8603, Japan; Department of Biological Chemistry, Graduate School of Pharmaceutical Sciences, Nagoya City University, Nagoya 467-8603, Japan; Department of Biological Chemistry, Graduate School of Pharmaceutical Sciences, Nagoya City University, Nagoya 467-8603, Japan

## Abstract

Stress induces global stabilization of the mRNA poly(A) tail (PAT) and the assembly of untranslated poly(A)-tailed mRNA into mRNPs that accumulate in stress granules (SGs). While the mechanism behind stress-induced global PAT stabilization has recently emerged, the biological significance of PAT stabilization under stress remains elusive. Here, we demonstrate that stress-induced PAT stabilization is a prerequisite for SG formation. Perturbations in PAT length impact SG formation; PAT shortening, achieved by overexpressing mRNA deadenylases, inhibits SG formation, whereas PAT lengthening, achieved by overexpressing their dominant negative mutants or downregulating deadenylases, promotes it. PABPC1, which specifically binds to the PAT, is crucial for SG formation. Complementation analyses reveal that the PABC/MLLE domain of PABPC1, responsible for binding PAM2 motif-containing proteins, plays a key role. Among them, ataxin-2 is a known SG component. A dominant-negative approach reveals that the PAM2 motif of ataxin-2 is essential for SG formation. Notably, ataxin-2 increases stress sensitivity, lowering the threshold for SG formation, probably by promoting the aggregation of PABPC1-bound mRNA. The C-terminal region is responsible for the self-aggregation of ataxin-2. These findings underscore the critical roles of mRNA PAT, PABPC1 and ataxin-2 in SG formation and provide mechanistic insights into this process.

## Graphical Abstract

**Graphical Abstract:**
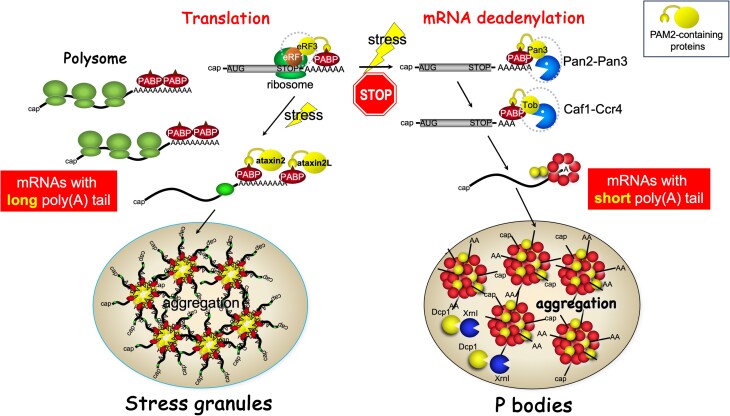

## Introduction

Stress granules (SGs) are cytoplasmic messenger ribonucleoprotein particles (mRNPs) formed when translation initiation is inhibited by environmental stress (1). Exposure of cells to environmental stress impairs translation initiation through the phosphorylation of the initiation factor eIF2α, inhibiting the formation of the eIF2-GTP-tRNAMet ternary complex and resulting in the disassembly of polysomes (2). Translationally repressed mRNPs accumulate within SGs, facilitated by self-aggregating proteins such as T-cell-restricted intracellular antigen-1 (TIA-1), TIA-1 related protein (TIAR) (1), and GTPase-activating protein SH3 domain-binding protein (G3BP) (3). SGs serve as mRNA storage sites protecting transcripts from environmental stress.

In addition to these key factors, SGs encompass various components, including PABPC1 (1), ataxin-2 (4,5), ataxin-2-like proteins (6) and ubiquitin specific peptidase 10 (USP10) (7,8). Ataxin-2 (*ATXN2*) is a causative gene in spinocerebellar ataxia type 2 (SCA2), an inherited neurodegenerative disease caused by the expansion of the CAG trinucleotide repeat encoding polyglutamine (polyQ) within the *ATXN2* gene (9,10). Ataxin-2 contains a Like-Sm (Lsm) domain, facilitating its binding to RNA oligonucleotides (11,12). Similarly, ataxin-2 related protein, ataxin-2-like, is an SG component sharing an Lsm domain with ataxin-2 but lacking polyglutamine residues (6). Employing a series of differential centrifugations, affinity purification of GFP-G3BP, and mass spectrometric analysis revealed the first stress granule proteome in mammalian cells (13). This proteome is notably enriched in translation factors, RNA-binding proteins, proteins featuring predicted prion-like domains, and those associated with neurodegenerative diseases. Transcriptome analysis, coupled with single-molecule fluorescence *in situ* hybridization (smFISH) validation, has demonstrated that essentially every mRNAs (∼10% of bulk mRNA molecules) and some non-coding RNA molecules accumulate within SGs although the targeting efficiency varies from <1% to >95% depending on the RNA molecules (14).

In addition to SGs, eukaryotic cells have another cytoplasmic foci known as processing bodies (P-bodies or PBs). PBs contain components such as decapping enzyme Dcp1/Dcp2, decapping activators DDX6, Lsm1-7, and the 5′-3′ exonuclease Xrn1, where mRNA decapping and degradation are thought to take place (15–17). Previous studies have identified deadenylase complexes Caf1–Ccr4 and Pan2–Pan3 in PBs, highlighting the significance of deadenylation for PB formation (18). Furthermore, PBs encompass mRNAs and rely on untranslated mRNAs for their assembly (19–21). Similar to PBs, SGs are regarded as mRNA storage sites; however, the intricate relationship between SG formation and mRNA deadenylation, and the role of poly(A) tail (PAT) length regulation in SG formation remains a topic of limited understanding.

In this study, we demonstrate the essential role of mRNA PAT in SG formation. The RNA-binding protein PABPC1, which specifically binds to the PAT, acts as a scaffold for SG assembly by recruiting the SG component ataxin-2. The C-terminal region of ataxin-2 facilitates its self-aggregation and the localization of the mRNP complex within SGs. These findings provide mechanistic insights into SG formation.

## Materials and methods

### Plasmids

To construct pCMV-5 × Myc-Pan2, pFlag-CMV5-Pan2 (22) was digested with EcoRI and SalI, and the Pan2 cDNA fragment was inserted into the EcoRI and SalI sites of pCMV-5 × Myc (23). To construct pCMV-5 × Flag-PABPC1, pCMV-5 × Myc-PABPC1, pCMV-5 × Flag-PABPC1 F567A/K580A/L585A, and pCMV-5 × Myc-PABPC1 F567A/K580A/L585A, PABPC1 and PABPC1 F567A/K580A/L585A cDNAs were PCR-amplified using the primer pair NH751/NH348 and pFlag-CMV5-PABPC1 and pFlag-CMV5-PABPC1 F567A/K580A/L585A (22), respectively, as a template. The resulting fragments were digested with SalI and inserted into the EcoRV and XhoI sites of pCMV-5 × Flag (24) and pCMV-5 × Myc, respectively. pCMV-5 × Myc-PABPC1 RNAi-resistant and pCMV-5 × Myc-PABPC1 F567A/K580A/L585A RNAi-resistant were generated by inverse PCR using pCMV-5 × Myc-PABPC1 and pCMV-5 × Myc-PABPC1 F567A/K580A/L585A, respectively, as a template and the primer pair NH554 /NH555. For the construction of pCMV-5 × Flag-ataxin-2 (1-458), ataxin-2 (1-79) and ataxin-2 (71–458) cDNAs were PCR-amplified using the HeLa cDNA library as a template and the primer pairs NH116/NH266 and NH258/NH262, respectively. To generate ataxin-2 (1–458) cDNA, ataxin-2 (1–79) was joined to ataxin-2 (71–458) using in-fusion PCR. The resulting fragment was digested with HindIII and XhoI and inserted into the HindIII and XhoI sites of pCMV-5 × Flag (23). To construct pCMV-Flag-ataxin-2, ataxin-2 (459–1313) cDNA was PCR-amplified using the primer pair NH112/NH113 and BC111757 clone as a template. The resulting fragment was digested with XhoI and SalI and inserted into the XhoI and SalI sites of pCMV-5 × Flag-ataxin-2 (1–458). pCMV-Flag-ataxin-2 F921A was generated by inverse PCR using pCMV-5 × Flag-ataxin-2 as a template and the primer pair NH134/NH135. To construct pCMV-5 × Myc-ataxin-2 and pCMV-5 × Myc-ataxin-2 F921A, pCMV-5 × Flag-ataxin-2 and pCMV-5 × Flag-ataxin-2 F921A were digested with HindIII and SalI, respectively, and ataxin-2 and ataxin-2 F921A cDNA fragments were inserted into the HindIII and SalI sites of pCMV-5 × Myc. pCMV-5 × Flag-ataxin-2 (1–240), pCMV-5 × Flag-ataxin-2 (1–548), pCMV-5 × Flag-ataxin-2 (1–905) and pCMV-5 × Flag-ataxin-2 (906–1313) were generated by inverse PCR using pCMV-5 × Flag-ataxin-2 as a template and the primer pairs NH145/RY138, NH0145/RY139, NH0145/RY140 and　RY137/NH144, respectively. To construct CMV-5 × Flag-GST-ataxin-2 (906–1313), pCMV-5 × Flag-GST-ataxin-2 (906–1095) and pCMV-5 × Flag-GST-ataxin-2 (1096–1313), ataxin-2 (906–1313), ataxin-2 (906–1095) and ataxin-2 (1096–1313) cDNA fragments were amplified using pCMV-5 × Flag-ataxin-2 as a template and the primer pairs HT012/HT18, HT015/HT020 and HT018/HT020, respectively. The resulting fragment was digested with EcoRI and SalI and inserted into the EcoRI and SalI sites of pCMV-Flag-GST (25). pCMV-5 × Flag-GST-ataxin-2 (925–1095) was generated by inverse PCR using pCMV-5 × Flag-GST-ataxin-2 (906–1095) as a template and the primer pair HT010/HT027. pCMV-5 × Flag-GST-ataxin-2 (925–1079) and pCMV-5 × Flag-GST-ataxin-2 (925–1050) were generated by inverse PCR using pCMV-5 × Flag-GST-ataxin-2 (925–1095) as a template and the primer pairs HT025/HT028 and HT026/HT028. To construct pCMV-Flag-ataxin-2-like, the open reading frame (ORF) of ataxin-2-like was amplified by PCR using the primer pair NH219/NH220 and BC082760 clone as a template. The resulting fragment was digested with EcoRI and inserted into the EcoRI site of pCMV-Flag. pCMV-Flag-ataxin-2-like F666A was generated by inverse PCR using pCMV-Flag-ataxin-2-like as a template and the primer pair NH248/NH249. To construct pBK-5 × Flag-EGFP, 5 × Flag-EGFP cDNA was PCR-amplified using pCMV-5 × Flag-EGFP (23) as a template and the primer pair NH733/NH732. The resulting fragment was digested with XhoI and EcoRI and inserted into XhoI and EcoRI sites of pBluescript II SK(–). To construct pBK-5 × Flag-pA30, EGFP cDNA was deleted by digestion with HindIII and self-ligation of the remaining plasmid. To construct pBK-5 × Flag-EGFP-pA72, β-globin 3′UTR with 72-base poly(A) cDNA was PCR-amplified using pFlag-CMV5/TO-BGG, a tetracycline-inducible β-globin expression vector, (22) as a template and the primer pair NH734/NH770. The resulting fragment was digested with EcoRI and inserted into the EcoRI and blunted-BamHI sites of pBK-5 × Flag-EGFP. The construction of all the other plasmids used in this study has been described previously (22,23,26). The synthetic oligonucleotides used in this study are listed in Supplementary Table S1. All genes used in this study are human origin.

### Cell culture and DNA/RNA transfection

HeLa cells were cultured in Dulbecco's modified Eagle's medium (Nissui) supplemented with 5% fetal bovine serum. DNA/RNA transfection was performed using Lipofectamine 2000 (Invitrogen), Polyethylenimine Max (Polyscience, Inc.) or Lipofectamine RNAi Max (Invitrogen) as previously described (26,27). For SG induction, HeLa cells were typically treated with arsenite (Wako) at a concentration of 0.5 mM for 30 min. To examine the dose-dependent effect of arsente on SG formation, cells were treated with the specified concentrations of arsenite for 30 min. PBs were analyzed in steady state conditions without the addition of arsenite.

### siRNA

The sequences of the other siRNAs used in this study are listed in Supplementary Table S2.

### Preparation of 5×flag-EGFP-poly(A)_0_ mRNA and 5×flag-EGFP-poly(A)_72_ mRNA.

pBK-5F-EGFP-poly(A)_72_, a template for *in vitro* mRNA transcription, was constructed as follows: EGFP cDNA was PCR-amplified using the primer pair NH733/NH732 and p5 × Flag-EGFP (23) as a template, and inserted into the XhoI and EcoRI sites of pBluscript II SK(-) to construct pBK-5 × Flag-EGFP. β-globin 3′-UTR, which contains a 72-nt poly(A) tail, was then PCR-amplified using the primer pair NH734/NH735 and pFlag-CMV/TO-BGG (22) as a template and inserted into EcoRI and BglⅡ sites of pBK-5 × Flag-EGFP to generate pBK-5F-EGFP-poly(A)_72_. pBK-5F-EGFP-poly(A)_0_, which contains no poly(A) tail, was obtained from pBK-5F-EGFP-poly(A)_72_ by artificial homologous recombination.

### Antibodies

The antibodies used in this study were anti-Flag (M2, Sigma), anti-Myc (9E10, Roche), anti-ataxin-2 (clone 22, BD Biosciences), anti-G3BP1 (clone 23, BD Biosciences), and anti-eIF3B (N-20, Santa Cruz Biotechnology). Anti-GAPDH, anti-PABPC1, and anti-ataxin-2-like antibodies were raised against the Hiss-tagged GAPDH, PABPC1, and ataxin-2-like, respectively. The anti-Caf1 antibody was a kind gift from Dr Ann-Bin Shyu (28).

### Immunofluorescence analysis

HeLa cells were grown on cover glass in Dulbecco's modified Eagle's medium supplemented with 5% fetal bovine serum. The transfected cells were washed once with phosphate buffered saline (PBS) and fixed with 4% paraformaldehyde in PBS for 10 min at room temperature. After quenching with 10 mM Glycine in PBS, the fixed cells were permeabilized with 0.1% Triton X-100 and 1% goat serum or 1% horse serum in PBS and incubated at 4°C overnight with appropriate primary antibodies. The cells were then washed four times with PBS and incubated at 4°C overnight with appropriate secondary antibodies. After four washes with PBS, the cells were mounted using ProLong Gold (Invitrogen). Images were captured using an OLYMPUS IX71 fluorescence microscope (Olympus, Tokyo, Japan). The SGs were detected using anti-G3BP1 or anti-eIF3B antibodies. The PBs were detected using an anti-DDX6 antibody. For quantitative analysis, 30 cells were analyzed for each experiment and the total intensity, number of cells, and size of SG and PB were measured using MetaMorph software (Molecular Devices) or cellSens software (Olympus). The secondary antibodies used for immunofluorescence analysis were Alexa Fluor 568-conjugated anti-mouse IgG, Alexa Fluor 488-conjugated anti-mouse IgG, Alexa Fluor 568-conjugated anti-rabbit IgG, Alexa Fluor 488-conjugated anti-rabbit IgG, Alexa Fluor 350-conjugated anti-goat IgG, and Alexa-568-conjugated anti-goat IgG (Invitrogen).

### Immunoprecipitation

The transfected cells were lysed in buffer A consisting of 50 mM Tris–HCl (pH 7.5), 50 mM NaCl, 0.1% Nonidet *P*-40, 1 mM dithiothreitol, 1 mM EDTA-Na (pH 8.0), 100 μM PMSF, 2 μg/ml of aprotinin, 2 μg/ml of Leupeptin and 10 μg/ml RNase A (Sigma) for 30 min on ice. After centrifugation at 15 000 × g for 20 min, the lysate was incubated for 1 h at 4°C with anti-Flag-agarose (Sigma). For the immunoprecipitation assay using the anti-PABPC1 antibody, the lysate was incubated with either anti-PABPC1 antibody or preimmune serum, and Protein G Sepharose 4 Fast Flow (GE Healthcare). The resin was then washed three times with buffer A. The bound protein was eluted using SDS-PAGE sample buffer and analyzed by western blotting.

### Isolation of soluble and insoluble cell fractions

HeLa cells were lysed in RIPA buffer consisting of 50 mM Tris–HCl (pH 7.5), 150 mM NaCl, 1% Triton-X-100, 0.1% SDS and 1% sodium deoxycholate for 15 min. The soluble and insoluble fractions were isolated by centrifugation at 15 000 × g for 20 min.

### RNA analysis

The total RNA isolation and northern blot analysis were performed as described previously (23,26).

### Statistical analysis

Statistical significance was determined using the Student's two-tailed unpaired *t*-test. For three or four samples, one three-way analysis of variance (ANOVA) with Bonferroni's multiple comparisons test was used.

## Results

### The long poly(A) tail (PAT) of mRNA promotes SG formation

**Figure 1.**
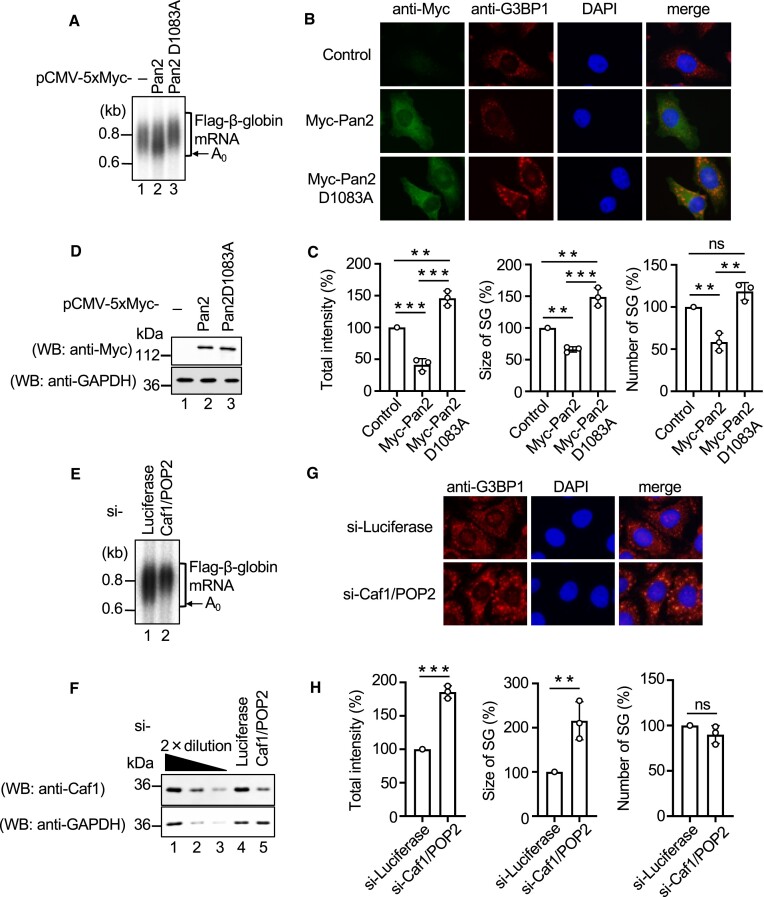
Poly (A) tail is required for SG formation. A to D, HeLa cells were co-transfected with the pFlag-CMV5/TO-BGG reporter plasmid and either pCMV-Myc, pCMV-5 × Myc-Pan2 or pCMV-5 × Myc-Pan2 D1083A. (**A**) β-globin mRNA was detected by northern blot analysis. (**B**) HeLa cells were treated with arsenite (0.5 mM) for 30 min and exogenous Pan2 and endogenous G3BP1 were detected by indirect immunofluorescence. (**C**) For quantitative analysis, the total intensity, size and number of SGs were calculated based on (B) using MetaMorph software. The quantitative value of SGs in the control cells was defined as 100%. (**D**) Proteins were analyzed by western blotting using the indicated antibodies. (E–H) HeLa cells were co-transfected with the pFlag-CMV5/TO-BGG reporter plasmid and luciferase siRNA or Caf1/POP2 siRNA. (**E**) β-globin mRNA was detected by northern blot analysis. (**F**) Proteins were analyzed by western blotting using the indicated antibodies. The leftmost three lanes, which analyzed 2-fold dilutions of HeLa cells extract, show that the conditions used for western blotting are semi-quantitative. (**G**) HeLa cells were treated with arsenite (0.5 mM) for 30 min, and endogenous G3BP1 was detected by indirect immunofluorescence. (**H**) For quantitative analysis, the total intensity, size and number of SGs were calculated based on Figure 1G using MetaMorph software. The quantitative value of SGs in luciferase siRNA-transfected cells was defined as 100%. Results are the average of three independent experiments and shown as means ± SD. **P < 0.01; ***P < 0.001.

Stress-induced stabilization of mRNA PAT and the formation of SGs are hallmarks of the cellular stress response. Because SGs consists of poly(A) plus mRNA, we surmised that the PAT, stabilized by the stress, might be involved in the formation of SGs. To test this hypothesis, we examined the impact of alterations in PAT length on SG formation. For this purpose, we perturbed the length of the mRNA PATs by either overexpressing or downregulating mRNA deadenylases. First, we investigated the effect of the overexpression of one of the major mRNA deadenylases, Pan2, on mRNA PAT length and SG formation. HeLa cells were co-transfected with plasmids expressing Flag-β-globin mRNA and either 5 × Myc-Pan2 or 5 × Myc-Pan2 D1083A, which has no deadenylase activity and acts as dominant-negative to the Pan2 deadenylase activity (22), and the mRNA was analyzed by northern blotting. To induce SG formation, cells were treated with sodium arsenite for 30 min, and SGs were detected by indirect immunofluorescence using an antibody against G3BP1 as an SG marker. Overexpression of wild-type Pan2 increased the amount of mRNAs with short PATs (Figure 1A, compare lanes 1 and 2). Under this condition, the total intensity, size and number of SGs decreased almost 2-fold (Figures 1B and 1C). In sharp contrast, overexpression of Pan2 D1083A mutant increased the amount of mRNAs with long PATs (Figure 1A, compare lanes 1 and 3), and the total intensity and size of SGs were increased almost 1.5-fold (Figure 1B and C). Expression of Pan2 and Pan2 D1083A was confirmed by western blotting (Figure 1D). In order to further examine the relationship between PAT length and SG formation, we utilized tetracycline-inducible expression system. T-REx-HeLa cells were co-transfected with pCMV-2 × TO-5 × Myc-Pan2 and a plasmid expressing 5 × Flag-β-globin reporter mRNA. Twenty-four hours after transfection, cells were treated with 100 ng/ml tetracycline to induce Pan2 production. The expression of 5 × Myc-Pan2, PAT shortening of the reporter mRNA and SG formation were monitored (Supplementary Figure S1). Time-dependent induction of 5 × Myc-Pan2 and shortening of the reporter mRNA PAT were observed after tetracycline treatment (Supplementary Figure S1A–C). During this time period, decreases in the total intensity, size and number of SGs were observed in a manner correlative to the shortening of the PAT (Supplementary Figure S1D and E).

Next, we examined the effect of overexpression of another major mRNA deadenylase, Caf1, on mRNA PAT length and SG formation. Cells were co-transfected with plasmids expressing Flag-β-globin mRNA and either 5 × Myc-Caf1 or 5 × Myc-Caf1 D161A, which has no deadenylase activity and acts as dominant-negative to the Caf1 deadenylase activity (22), and the PAT length of the mRNA and SG formation were analyzed as above. Overexpression of wild-type Caf1 increased the amount of mRNAs with short PATs (Supplementary Figure S2A, lanes 1 and 2), and the total intensity, size and number of SGs decreased by almost 2-fold (Supplementary Figure S2B and C). In contrast, the overexpression of Caf1 D161A increased the amount of mRNAs with long PATs (Supplementary Figure S2A, lanes 1 and 3), and the total intensity, size and number of SGs were increased (Supplementary Figure S2B and C). Caf1 and Caf1 D161A expression was confirmed by western blotting (Supplementary Figure S2D).

We also examined the effect of deadenylase downregulation. Cells were co-transfected with a plasmid expressing Flag-β-globin mRNA and either control or Caf1/POP2 siRNAs, and the PAT length of Flag-β-globin mRNA and SG formation were analyzed as described above. Caf1 knockdown increased the amount of mRNAs with long PATs (Figure 1E). The expression of Caf1 was confirmed using western blotting (Figure 1F). The total intensity and size of SGs (Figures 1G and H) increased almost 2-fold. Finally, we confirmed whether the PAT directly promoted SG formation. Cells were transfected with *in vitro* synthesized 5 × Flag-EGFP-poly(A)_0_ mRNA and 5 × Flag-EGFP-poly(A)_72_ mRNA. Transfected mRNAs were confirmed using northern blotting (Supplementary Figure S2E). Only 5 × Flag-EGFP-poly(A)_72_ mRNA promoted SG formation (Supplementary Figure S2F and G). Collectively, these results indicate that SG formation is compromised when the mRNA PAT is shortened whereas SG formation is promoted when the mRNA PAT is lengthened, suggesting that the PAT plays an important role in SG formation.

### The short poly(A) tail (PAT) of mRNA promotes P body formation

Similar to SGs, another cytoplasmic foci, PBs, also contain translationally repressed mRNAs. However, in contrast to the above results, showing that SG formation is suppressed when the mRNA PAT length is shortened by deadenylase overexpression, a previous study showed that deadenylation is a prerequisite for PB formation (18). Thus, we speculate that PB formation, as opposed to SG formation, is promoted when the mRNA PAT length is short. To test this hypothesis, we examined the effects of deadenylase overexpression and downregulation on PB formation. We expressed 5 × Myc-Pan2, 5 × Myc-Pan2 D1083A mutant, 5 × Myc-Caf1, or 5 × Myc-Caf1 D161 mutant and analyzed PB formation by indirect immunofluorescence using the PB marker protein DDX6. When the mRNA PATs were shortened by the overexpression of wild-type Pan2 and Caf1, the total intensity, size, and number of PBs increased. In sharp contrast, when the mRNA PATs were lengthened by the overexpression of Pan2 D1083A and Caf1 D161 mutants, the total intensity, size, and number of PBs decreased (Supplementary Figure S3A–D). Caf1 knockdown decreased the intensity, size and number of PBs (Supplementary Figure S3E and F). These results suggest that the mRNA PAT length exerts a negative impact on PB formation, in sharp contrast to SG formation.

### The PABC/MLLE domain of PABPC1 is required for SG formation.

**Figure 2.**
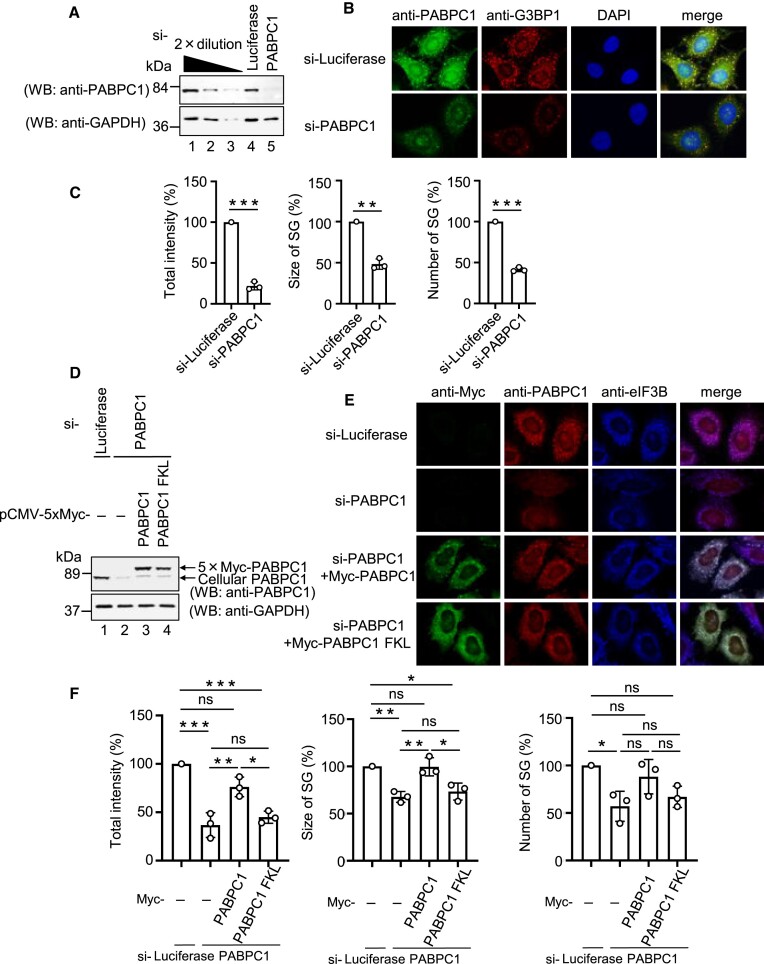
PABC/MLLE domain of PABPC1 is required for SG formation. A to C, HeLa cells were transfected with luciferase siRNA or PABPC1 siRNA. (**A**) Proteins were analyzed by western blotting using the indicated antibodies. The leftmost three lanes, which analyzed 2-fold dilutions of HeLa cells extract, show that the conditions used for western blotting are semi-quantitative. (**B**) HeLa cells were treated with arsenite (0.5 mM) for 30 min, and endogenous PABPC1 and G3BP1 were detected by indirect immunofluorescence. (**C**) For quantitative analysis, the total intensity, size, and number of SGs were calculated based on (B) using MetaMorph software. The quantitative value of SGs in the control cells was defined as 100%. (D–F) HeLa cells were transfected with luciferase siRNA or PABPC1 siRNA. At the 24 h after siRNA transfection, the cells were transfected with pCMV-Myc, pCMV-5 × Myc-PABPC1 RNAi-resistant or pCMV-5 × Myc-PABPC1-FKL (F567A/K580A/L585A) RNAi-resistant. (**D**) Proteins were analyzed by western blotting using the indicated antibodies. (**E**) HeLa cells were treated with arsenite (0.5 mM) for 30 min and exogenous PABPC1, endogenous PABPC1, and endogenous eIF3B were detected by indirect immunofluorescence. (**F**) For quantitative analysis, the total intensity, size, and number of SGs were calculated based on (E) using MetaMorph software. The quantitative value of SGs in luciferase siRNA-transfected cells was defined as 100%. Results are the average of three independent experiments and shown as means ± SD. *P < 0.05; **P < 0.01; ***P < 0.001.

SGs contain a general mRNA-binding protein, PABPC1, which plays a versatile role in mRNA metabolism by binding to the PAT. Thus, we hypothesized that PABPC1 plays a role in SG formation. To test this hypothesis, we examined the effect of PABPC1 downregulation on SG formation. Protein expression was confirmed using western blotting (Figure 2A). siRNA-mediated knockdown of PABPC1 significantly inhibited SG formation (Figure 2B and C). These results indicate that PABPC1 is necessary for SG formation.

We and others have previously shown that PABPC1 plays an essential role in PAT metabolism by binding to the PAM2 motif-containing proteins, Tob (22,24,28,29), Pan3 (22,30), eRF3 (22,31–33) and ataxin-2 (5,34–37), via its PABC/MLLE domain. Therefore, we investigated whether the PABC/MLLE domain of PABPC1 is required for SG formation. To test this hypothesis, we used an siRNA rescue experiment. After cells were depleted of PABPC1 by siRNA transfection, they were again transfected with a plasmid expressing either siRNA-resistant 5 × Myc-PABPC1 mRNA or siRNA-resistant 5 × Myc-PABPC1-FKL mRNA, which contains mutations (F567A/K580A/L585A) in PABC/MLLE domain and the protein product cannot bind to PAM2 motif-containing proteins (22). SGs were detected by indirect immunofluorescence using the SG marker protein eIF3B. The expression of PABPC1 was confirmed by western blotting (Figure 2D). When SG formation was inhibited by the depletion of PABPC1, SG formation was rescued by wild-type PABPC1 expression (Figure 2E and F). In contrast, PABPC1-FKL had no significant effect (Figure 2E and F). These results indicated that the PABC/MLLE domain of PABPC1 is required for SG formation.

### The PAM2 motif of ataxin-2 is required for SG formation

Because PABPC1 regulates SG formation through its PABC/MLLE domain, we hypothesized that poly(A)-bound PABPC1 regulates SG formation by binding to PAM2 containing proteins. Ataxin-2 and ataxin-2-like proteins are known as SG components as well as PAM2 motif containing proteins (5,6,36). Therefore, we determined whether both proteins bind to the PABC/MLLE domain of PABPC1 via their PAM2 motifs. First, we examined the interactions between endogenous proteins. HeLa cell extracts were immunoprecipitated using an anti-PABPC1 antibody. Both ataxin-2 and ataxin-2-like proteins coprecipitated with PABPC1 (Supplementary Figure S4A) as previously described (5,6). Next, we examined whether PABPC1 binds to ataxin-2 and ataxin-2-like proteins through its PABC/MLLE domain. Cells were transfected with a plasmid expressing 5 × Flag-PABPC1 or 5 × Flag-PABPC1-FKL, and cell extracts were immunoprecipitated with an anti-Flag antibody. Endogenous ataxin-2/ ataxin-2-like proteins co-precipitated with 5 × Flag-PABPC1, but not with 5 × Flag-PABPC1-FKL (Supplementary Figure S4B). Finally, we analyzed the interaction between ataxin-2/ ataxin-2-like with loss-of-function mutation in PAM2 motif (ataxin-2 F921A and ataxin-2-like F666A) (33) and endogenous PABPC1. Either Flag-ataxin-2, Flag-ataxin-2 F921A, Flag-ataxin-2-like or Flag-ataxin-2-like F666A were expressed in the cells, and immunoprecipitation assays were performed as described above. The binding of ataxin-2/ ataxin-2-like with PABPC1 was abolished by the mutation of their PAM2 motifs (Supplementary Figure S4C and D). These results indicated that ataxin-2 and ataxin-2-like proteins bind to the PABC/MLLE domain of PABPC1 through their PAM2 motifs.

**Figure 3.**
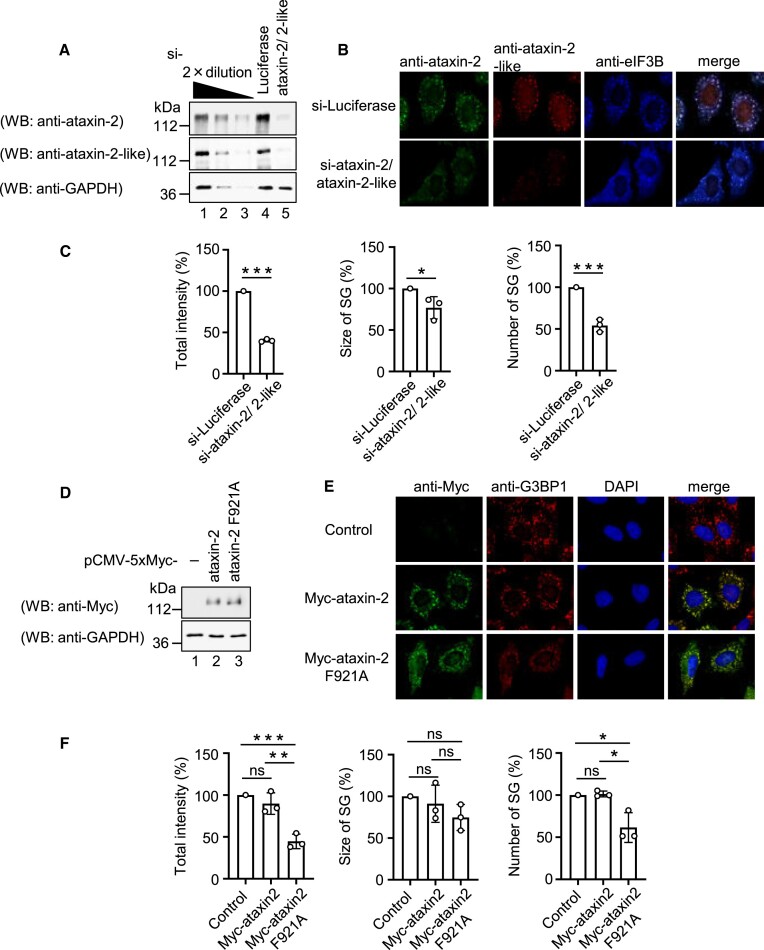
PAM2 motif of ataxin-2 is required for SG formation. (A–C) HeLa cells were transfected with luciferase siRNA or ataxin2/ataxin2-like siRNA. (**A**) Proteins were analyzed by western blotting using the indicated antibodies. The leftmost three lanes, which analyzed 2-fold dilutions of HeLa cells extract, show that the conditions used for western blotting are semi-quantitative. (**B**) HeLa cells were treated with arsenite (0.5 mM) for 30 min, and endogenous ataxin-2, endogenous ataxin-2-like, and endogenous eIF3B were detected by indirect immunofluorescence. (**C**) For quantitative analysis, the total intensity, size, and number of SGs were calculated based on (B) using MetaMorph software. The quantitative value of SGs in luciferase siRNA transfected cells was defined as 100%. (D–F) HeLa cells were transfected with pCMV-Myc, pCMV-5 × Myc-ataxin-2, or pCMV-5 × Myc-ataxin-2 F921A. (**D**) Proteins were analyzed by western blotting using the indicated antibodies. (**E**) HeLa cells were treated with arsenite (0.5 mM) for 30 min, and exogenous ataxin-2 and endogenous G3BP1 were detected using indirect immunofluorescence. (**F**) For quantitative analysis, the total intensity, size, and number of SGs were calculated based on (E) using MetaMorph software. The quantitative value of SGs in the control cells was defined as 100%. Results are the average of three independent experiments and shown as means ± SD. *P < 0.05; **P < 0.01; ***P < 0.001.

Based on these results, we investigated whether ataxin-2 regulates SG formation by binding to PABPC1. We examined the effects of ataxin-2 and ataxin-2-like proteins on SG formation by using knockdown strategy. The expressions of ataxin-2 and ataxin-2-like proteins was confirmed by western blotting (Figure 3A). Knockdown of ataxin-2 and ataxin-2-like proteins repressed SG formation (Figure 3B and C), which is consistent with previous observation by the Krobitsch group (4). Next, we overexpressed the ataxin-2 PAM2 motif mutant in cells, and analyzed SG formation. The expression of ataxin-2 was confirmed using western blotting (Figure 3D). Overexpression of the ataxin-2 PAM2 motif mutant repressed SG formation in a dominant-negative manner, where wild-type ataxin-2 had no significant effect (Figure 3E and F). These results indicated that the PAM2 motif of ataxin-2/ ataxin-2 like proteins is required for SG formation. Collectively, these results suggest that ataxin-2/ataxin-2 like proteins bind to the PABC/MLLE domain of PABPC1 via their PAM2 motifs to regulate SG formation.

### Ataxin-2 promotes sensitivity to arsenite stress in SG formation

**Figure 4.**
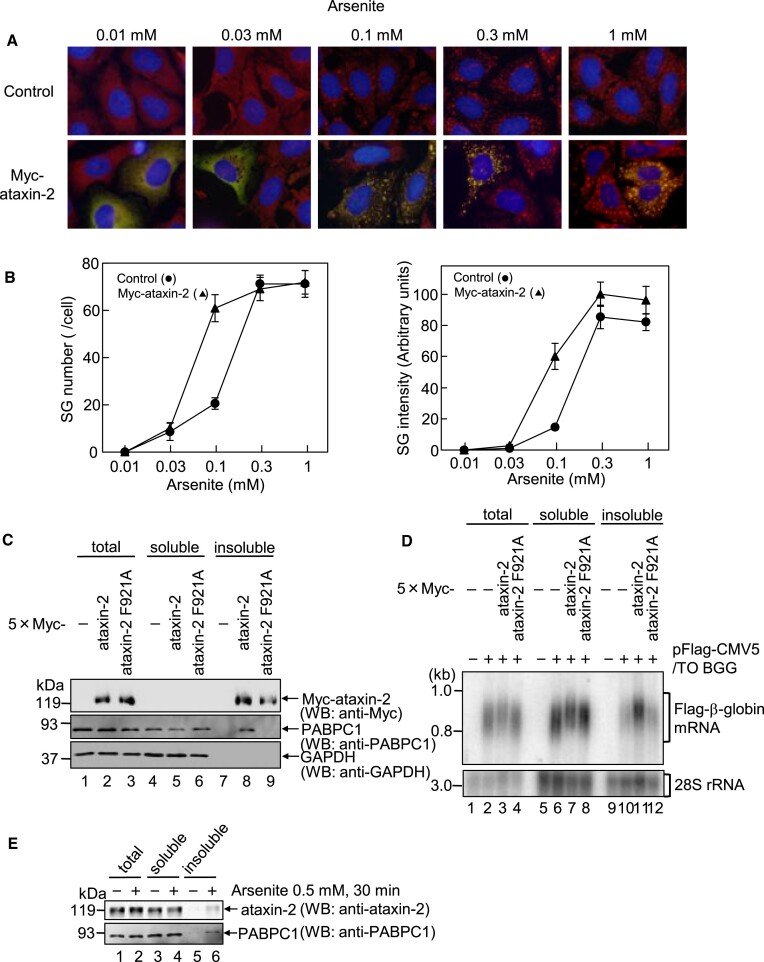
Ataxin-2 promotes responsiveness in SG formation. (**A**) HeLa cells that were transfected with either pCMV-Myc or pCMV-5 × Myc-ataxin-2 were treated with the specified concentrations of arsenite for 30 min. Myc-ataxin-2 and endogenous G3BP1 were detected using indirect immunofluorescence. (**B**) For quantitative analysis, the number (left) or total intensity (right) of SGs was calculated based on (A) using MetaMorph software. Results are the average of three independent experiments and shown as means ± SD. (**C**) HeLa cells were transfected with pCMV-Myc, pCMV-5 × Myc-ataxin-2, or pCMV-5 × Myc-ataxin-2 F921A. The soluble or insoluble fraction was isolated from the cells, and equal aliquots corresponding to the same amount of cells were analyzed by western blotting using the indicated antibodies. (**D**) HeLa cells were co-transfected with pFlag-CMV5/TO-BGG reporter plasmid and either pCMV-Myc, pCMV-5 × Myc-ataxin-2 or pCMV-5 × Myc-ataxin-2 F921A. Soluble and insoluble fractions were isolated from the cells and equal aliquots corresponding to the same amount of cells were analyzed by northern blotting using the specified probes. (**E**) HeLa cells were treated with 0.5 mM arsenite for 30 min. Soluble and insoluble fractions were isolated from the cells, and equal aliquots corresponding to the same amount of cells were analyzed by western blotting using the indicated antibodies.

To determine how ataxin-2 promotes SG formation, we examined the effect of ataxin-2 on the dose-dependent effect of arsenite on the formation of SGs. SGs were detected by indirect immunofluorescence using the SG marker protein eIF3B. SGs appeared at 10 uM concentration of arsenite and increased in a manner dependent on arsenite concentration, reaching a plateau at 0.3 mM. The dose-response curve was shifted to the left by the ectopic expression of Myc-ataxin-2 (Figure 4A and B). These results are consistent with the results in Figure 3D–F, in which SG formation was not affected by the expression of Myc-ataxin-2 at 0.5 mM arsenite. These results indicated that ataxin-2 increases sensitivity to arsenite stress during SG formation.

It is known that the self-aggregation of G3BP1 through its nuclear transport factor 2 (NTF2) domain is important for SG formation (3). Therefore, we hypothesized that ataxin-2 also promotes SG formation via protein aggregation. To assess ataxin-2 protein aggregation, HeLa cells were transfected with a plasmid expressing either 5 × Myc-ataxin-2 WT or 5 × Myc-ataxin-2 F921A, suspended in RIPA buffer, and centrifuged for sequential separation of soluble and insoluble fractions. The fractions were analyzed by western blotting. Both 5 × Myc-ataxin-2 and 5 × Myc-ataxin-2 F921A proteins were detected in the insoluble fraction (Figure 4C, lanes 8 and 9), suggesting that ataxin-2 can aggregate. Interestingly, PABPC1 was also detected in the insoluble fraction of ataxin-2 WT-expressing cells but not that in ataxin-2 F921A-expressing cells (Figure 4C, lanes 8 and 9), indicating that ataxin-2 promotes the aggregation of PABPC1 protein via the PAM2 motif. In addition, to analyze mRNA aggregation, HeLa cells were co-transfected with pFlag-CMV5/TO-BGG reporter plasmid expressing β-globin mRNA and either 5 × Myc-ataxin-2 WT or 5 × Myc-ataxin-2 F921A. Similar to the PABPC1 protein, β-globin mRNA was also detected in the insoluble fraction of ataxin-2 WT-expressing cells, but not in ataxin-2 F921A-expressing cells (Figure 4D, lanes 11 and 12). These results indicate that ataxin-2 promotes the aggregation of the reporter mRNA via the PAM2 motif.

We also investigated whether arsenaite stress induces ataxin-2 aggregation. Cells were treated with sodium arsenite (0.5 mM) for 30 min, and then, the soluble and insoluble fractions were separated using RIPA buffer, as described above. Under normal conditions, endogenous ataxin-2 and PABPC1 were both detected in soluble fraction, whereas under arsenite-stressed conditions, both ataxin-2 and PABPC1 moved to the insoluble fraction (Figure 4E, lanes 5 and 6), suggesting that arsenite stress induces the aggregation of ataxin-2 and PABPC1.

### The ataxin-2 C-terminal 925-1079 aa region (C1 region) is required for its localization to SGs

**Figure 5.**
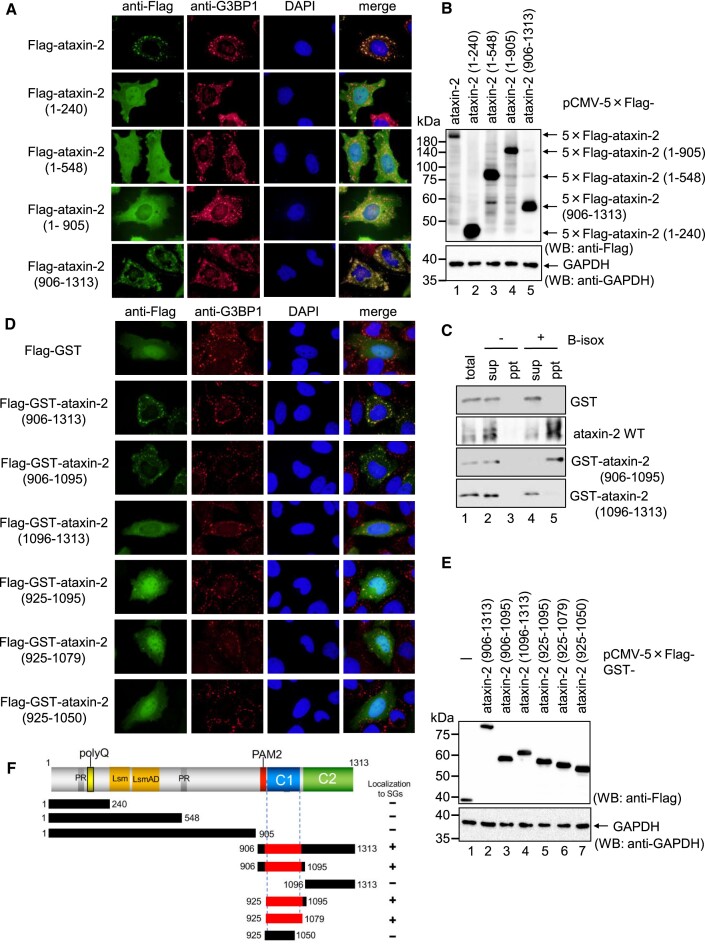
Ataxin-2 C-terminal 925–1079 aa region is required for its localization to SGs. (**A**) HeLa cells were transfected with pCMV-5 × Flag-ataxin-2, pCMV-5 × Flag-ataxin-2 (1–240), pCMV-5 × Flag-ataxin-2 (1–548), pCMV-5 × Flag-ataxin-2 (1–905) or pCMV-5 × Flag-ataxin-2 (906–1313). The cells were treated with 0.5 mM arsenite for 30 min, and exogenous ataxin-2 and endogenous G3BP1 were detected by indirect immunofluorescence. (**B**) Proteins were analyzed by western blotting using the indicated antibodies. (**C**) HeLa cells were transfected with pCMV-5 × Flag-GST, pCMV-5 × Flag-ataxin-2, pCMV-5 × Flag-GST-ataxin-2 (906–1095), or pCMV-5 × Flag-GST-ataxin-2 (1096–1313). The cells lysates were incubated with B-isox for 90 mins at 10°C and then the precipitate fraction was collected by centrifugation. The precipitate fraction and supernatant fraction were analyzed by western blotting. (**D**) HeLa cells were transfected with pCMV-5 × Flag-GST, pCMV-5 × Flag-GST-ataxin-2 (906–1313), pCMV-5 × Flag-GST-ataxin-2 (906–1095), pCMV-5 × Flag-GST-ataxin-2 (1096–1313), pCMV-5 × Flag-GST-ataxin-2 (925–1095), pCMV-5 × Flag-GST-ataxin-2 (925–1079), or pCMV-5 × Flag-GST-ataxin-2 (925–1050). The cells were treated with 0.5 mM arsenite for 30 min, and exogenous ataxin-2 and endogenous G3BP1 were detected by indirect immunofluorescence. (**E**) Proteins were analyzed by western blotting using the indicated antibodies. (**F**) Schematic diagram of ataxin-2 fragments with a summary of the ataxin-2 localization to SGs.

These results led us to hypothesize that ataxin-2 recruits not only PABPC1 but also poly(A)-tailed mRNA into SGs. To unravel what determines the localization of ataxin-2 to SGs, we first identified the region of ataxin-2 required for its localization to SGs. We constructed a series of deletion mutants of ataxin-2: ataxin-2 (1–240), ataxin-2 (1–548), ataxin-2 (1–905) and ataxin-2 (906–1313). The subcellular localization of the mutants was assessed after arsenite treatment for 30 min. Among these mutants, ataxin-2 (906–1313) localized to the SGs, as in the case of ataxin-2 WT (Figure 5A and Supplementary Table S3), whereas all C-terminal deletion mutants did not. Protein expression was confirmed using western blotting (Figure 5B). To further confirm that the ataxin-2 C-terminal region can localize to SGs, we utilized a biotinylated isoxazole (B-isox) fractionation assay. B-isox is a chemical reagent that selectively precipitates SG component proteins to form RNA granule-like aggregates (38,39). HeLa cells were transfected with a plasmid expressing either 5 × Flag-tagged GST, ataxin-2, ataxin-2 (906–1095) or ataxin-2 (1096–1313). Cell lysates were incubated with B-isox for 90 min at 10°C and the precipitated fraction was collected by centrifugation. The precipitate and supernatant were analyzed by western blotting. ataxin-2 (906–1095), as well as ataxin-2 WT (positive control), were detected in the precipitates (Figure 5C, lane 5), whereas ataxin-2 (1096–1313) was detected in the supernatants (Figure 5C, lane 4). These results indicate that the 906–1095 aa region is localized to SGs.

To further narrow down the region of ataxin-2 required for localization to SGs, we constructed deletion mutants based on GST-fused ataxin-2 (906–1313). Cells were transfected with a plasmid expressing 5 × Flag-tagged GST or GST-fused ataxin-2 deletion mutants, and their subcellular localization was analyzed after treatment with arsenite for 30 mins. GST-ataxin-2 (906–1313) and GST-ataxin-2 (906–1095) were localized to the SGs, whereas GST-ataxin-2 (1096–1313) and GST alone were not (Figure 5D and Supplementary Table S4). Furthermore, although less efficient than GST-ataxin-2 (906–1313) and GST-ataxin-2 (906–1095), GST-ataxin-2 (925–1095) and GST-ataxin-2 (925–1079), which do not contain the PAM2 motif, but not ataxin-2 (925–1050), were localized to SGs (Figures 5D and Supplementary Table S4). Protein expression was confirmed using western blotting (Figure 5E). These results indicate that the ataxin-2 925–1079 aa region (hereafter denoted as the C1 region) is necessary for its localization to SGs (Figure 5F).

### The ataxin-2 C-terminal 1096-1313 aa region (C2 region) is required for its self-aggregation.

**Figure 6.**
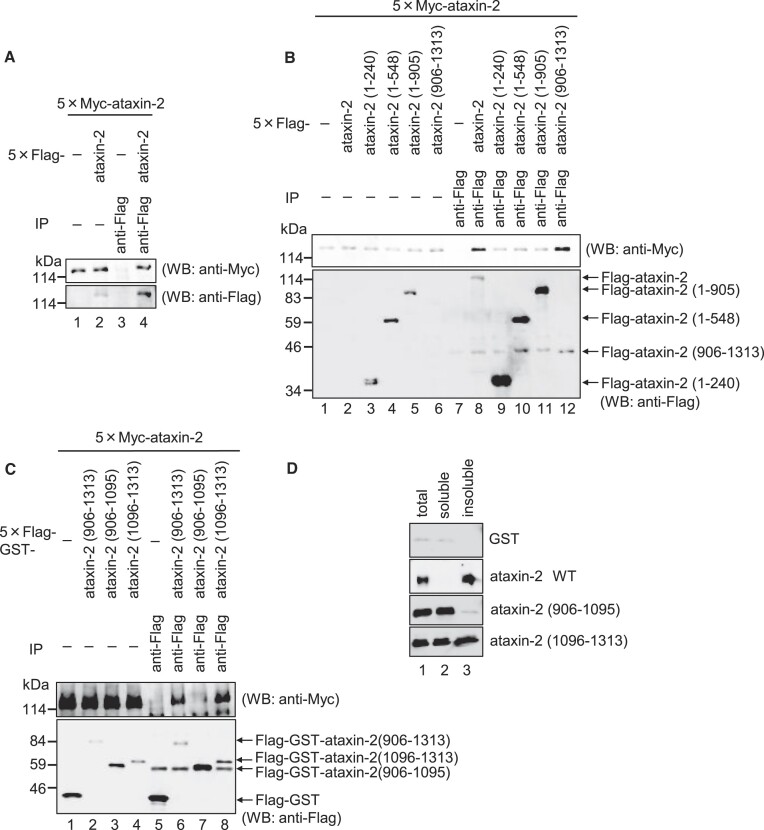
Ataxin-2 C-terminal 1096–1313 aa region is required for its self-aggregation. (**A**) HeLa cells were co-transfected with pCMV-5 × Myc-ataxin-2 and either pCMV-5 × Flag (lanes 1 and 3) or pCMV-5 × Flag-ataxin-2 (lanes 2 and 4). The cells extracts were subjected to an immunoprecipitation (IP) assay using anti-Flag antibody. The immunoprecipitation and inputs were analyzed by western blotting with the indicated antibodies. (**B**) HeLa cells were co-transfected with pCMV-5 × Myc-ataxin-2 and either pCMV-5 × Flag (lanes 1 and 7), pCMV-5 × Flag-ataxin-2 (lanes 2 and 8), pCMV-5 × Flag-ataxin-2 (1–240) (lanes 3 and 9), pCMV-5 × Flag-ataxin-2 (1–548) (lanes 4 and 10), pCMV-5 × Flag-ataxin-2 (1–905) (lanes 5 and 11), or pCMV-5 × Flag-ataxin-2 (906–1313) (lanes 6 and 12). The cells extracts were subjected to an IP assay and western blotting as described in (A). (**C**) HeLa cells were co-transfected with pCMV-5 × Myc-ataxin-2 and either pCMV-5 × Flag-GST (lanes 1 and 5), pCMV-5 × Flag-GST-ataxin-2 (906–1313) (lanes 2 and 6), pCMV-5 × Flag-GST-ataxin-2 (906–1095) (lanes 3 and 7), or pCMV-5 × Flag-GST-ataxin-2 (1096–1313) (lanes 4 and 8). The cell extracts were subjected to an IP assay and western blotting as described in (A). (**D**) HeLa cells were transfected with pCMV-5 × Flag-GST, pCMV-5 × Flag-ataxin-2, pCMV-5 × Flag-ataxin-2 (906–1095) or pCMV-5 × Flag-ataxin-2 (1096–1313). The soluble or insoluble fraction was isolated from the cells and analyzed by western blotting with the indicated antibodies.

Figure 4 suggests that ataxin-2 tends to aggregate, and its aggregation is induced by arsenite stress. Thus, we investigated whether self-aggregation of ataxin-2 is required for SG formation. We tested whether ataxin-2 can form a multimer. Cells were co-transfected with plasmids expressing 5 × Flag-ataxin-2 and 5 × Myc-ataxin-2 and cell lysates were subjected to immunoprecipitation with an anti-Flag antibody. As shown in Figure 6A, 5 × Myc-ataxin-2 co-precipitated with 5 × Flag-ataxin-2. Based on our finding that the C-terminal C1 region of ataxin-2 is important for its localization to SGs, we anticipated that ataxin-2 forms multimers through its C-terminal region. To test this hypothesis, we used the ataxin-2 deletion mutants described above (Figure 5A). After the cells were co-transfected with a plasmid expressing 5 × Myc-ataxin-2 and either 5 × Flag-ataxin-2, 5 × Flag-ataxin-2 (1-240), 5 × Flag-ataxin-2 (1-548), 5 × Flag-ataxin-2 (1-905) or 5 × Flag-ataxin-2 (906–1313), the cells extracts were subjected to immunoprecipitation with an anti-FLAG antibody. Consistent with this expectation, 5 × Myc-ataxin-2 co-precipitated with 5 × Flag-ataxin-2 (906–1313) as well as 5 × Flag-ataxin-2 (Figure 6B). To further explore the region required for multimer formation, we performed immunoprecipitation using the GST-fused ataxin-2 deletion mutants described above (Figure 5D). After the cells were co-transfected with a plasmid expressing 5 × Myc-ataxin-2 and either 5 × Flag-GST, 5 × Flag-GST-ataxin-2 (906–1313), 5 × Flag-GST-ataxin-2 (906–1095) or 5 × Flag-GST-ataxin-2 (1096–1313), the cell lysates were subjected to immunoprecipitation with an anti-FLAG antibody. 5 × Myc-ataxin-2 co-precipitated with 5 × Flag-GST-ataxin-2 (1096–1313) as well as 5 × Flag-GST-ataxin-2 (906–1313), but not with 5 × Flag-GST-ataxin-2 (906–1095) (Figure 6C). These results indicate that ataxin-2 forms a multimer in a region (1096–1313) separate from the C1 region (925–1079) required for SG localization.

Next, we investigated whether the formation of the ataxin-2 multimer led to its aggregation by assessing the aggregation capacity of the ataxin-2 C-terminal region. Cells expressing 5 × Flag-tagged ataxin-2, ataxin-2 (906–1095), ataxin-2 (1096–1313) or GST were suspended in RIPA buffer and centrifuged for the sequential separation of soluble and insoluble fractions. The fractions were analyzed by western blotting. As shown in Figure 6D, ataxin-2 was detected definitely in the insoluble fraction. In sharp contrast, ataxin-2 (906–1095) was detected in the soluble fraction, whereas ataxin-2 (1096–1313) was detected in both soluble and insoluble fractions (Figure 6D). Taken together, these results indicate that the ataxin-2 C-terminal 1096–1313 aa region (hereafter denoted as the C2 region) is required not only for multimer formation but also for the aggregation capacity of ataxin-2.

### Self-aggregation of ataxin-2 is required for SG formation.

**Figure 7.**
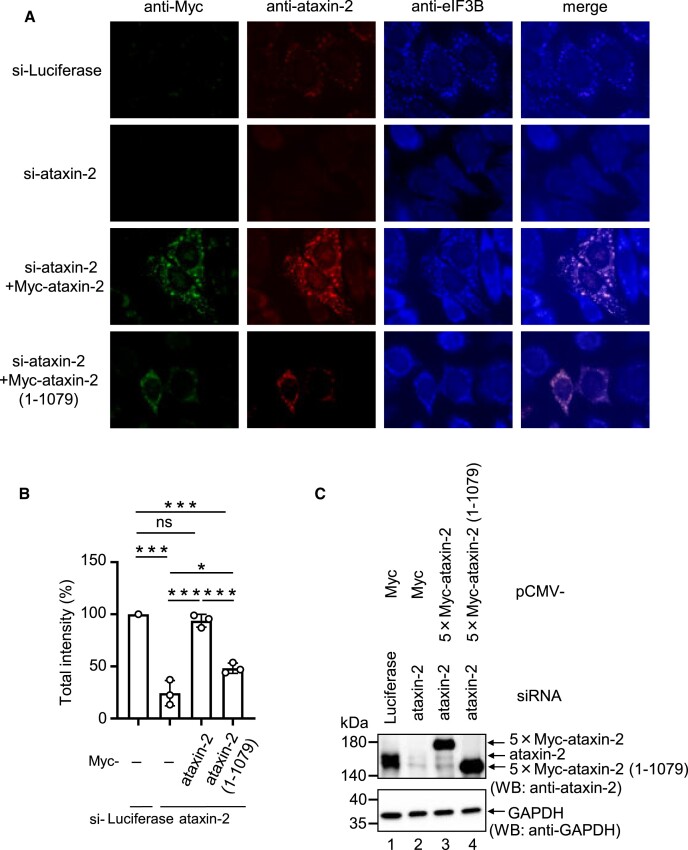
Self-aggregation of ataxin-2 is required for SG formation. (**A**) HeLa cells were transfected with luciferase siRNA or ataxin-2 siRNA. In the 24 h after siRNA transfection, cells were transfected with pCMV-Myc, pCMV-5 × Myc-ataxin-2 RNAi-resistant or pCMV-5 × Myc-ataxin-2 (1–1079) RNAi-resistant. The cells were treated with arsenite (0.5 mM) for 30 min, and exogenous ataxin-2, endogenous ataxin-2, and endogenous eIF3B were detected by indirect immunofluorescence. (**B**) For quantitative analysis, the total intensity of SGs was calculated based on Figure 7A using MetaMorph software. The quantitative value for SGs in control cells was defined as 100%. Results are the average of three independent experiments and shown as means ± SD. (**C**) Western blot showing expression of 5 × Myc-fused proteins and the knockdown efficiency. *P < 0.05; ***P < 0.001.

As mentioned above, the C2 region of ataxin-2 is relevant for its self-aggregation. Thus, to test whether the C2 region is required for SG formation, we performed a rescue experiment by examining the ability of the ataxin-2 C2-deletion mutant to rescue SG formation in ataxin-2 knockdown cells. After the cells were treated with either ataxin-2 siRNA or luciferase siRNA (control) for 24 h, they were transfected with a plasmid expressing either siRNA-resistant 5 × Myc-ataxin-2 mRNA or siRNA-resistant 5 × Myc-ataxin-2 (1–1079) mRNA. SGs were detected by indirect immunofluorescence using the SG marker protein eIF3B. The siRNA-mediated downregulation of ataxin-2 inhibited SG formation by ∼20%. Under these conditions, the expression of siRNA-resistant ataxin-2 almost completely recovered SG formation, whereas siRNA-resistant ataxin-2 (1–1079) compromised the ability to recover SG formation (Figure 7A and B). Protein expression was confirmed using western blotting (Figure 7C). These results suggest that the C2 region of ataxin-2, which is responsible for ataxin-2 self-aggregation, is required for SG formation.

## Discussion

**Figure 8.**
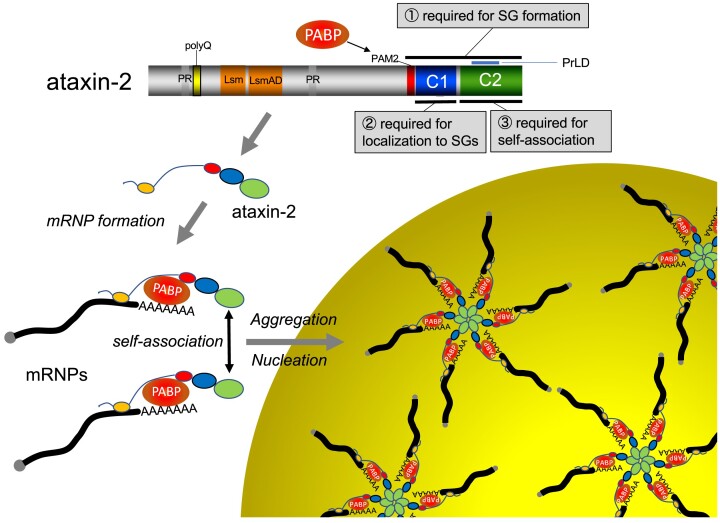
Model for the ataxin-2, PABPC1 and mRNA PAT-mediated SG formation. Under stress conditions, mRNAs are translationally silenced and mRNA deadenylation is globally ceased, which leads to the accumulation of mRNAs with long PATs. The PABPC1 bound to the PAT acts as a scaffold to recruit ataxin-2 via PABC-PAM2 contact. The C2 region of ataxin-2 triggers the aggregation of PABPC1 in complex with mRNAs, leading to the promotion of SG formation. mRNPs would actually contain numerous sites for potential interactions with other mRNPs through protein-protein or intermolecular RNA-RNA interactions, however, a more simplified illustration is provided based on the results of this study. For illustrative purpose, the mRNP complex is shown as a hexamer.

In this study, we investigated the mechanism underlying arsenite-stress-induced SG formation by focusing on mRNA poly(A) tail (PAT) metabolism. Here, we showed that (i) global shortening of the mRNA PAT by expressing Pan2 and Caf1 deadenylases inhibited SG formation (Figures 1, S1 and S2), (ii) global lengthening of the PAT by expressing dominant-negative mutants of deadenylases Pan2 D1083A and Caf1 D161A as well as by siRNA-mediated downregulation of Caf1/POP2 promoted SG formation, suggesting that the mRNA PAT is required for the formation of SGs (Figures 1, S1 and S2). Consistent with this idea, (iii) SG formation was compromised by siRNA-mediated downregulation of PABPC1, which specifically binds to the PAT (Figure 2A–C). The fact that PABPC1 with a mutation in the PABC/MLLE domain could not complement SG formation indicates that the PABC/MLLE domain is required for SG formation (Figure 2D–F). We also found that (iv) ataxin-2, which binds to the PABC/MLLE domain via its PAM2 motif, is required for SG formation (Figure 3). The PAM2 motif of ataxin-2 is a prerequisite for this effect (Figure 3D–F). (v) ataxin-2 increased the stress sensitivity (Figure 4A and B), probably through the accelerated formation of mRNP aggregates consisting of PABPC1 bound to the mRNA (Figure 4C–E). Finally, we identified a C-terminal region of 906–1095 aa (C1 region) required for its localization to SGs (Figure 5), and 1096–1313 aa (C2 region) required for self-aggregation of ataxin-2 (Figure 6). From these results, we propose a model for SG formation (Figure 8). Under stress conditions, mRNAs are translationally silenced and mRNA deadenylation is globally ceased. Thus, mRNAs with long PATs accumulate (40). The PABPC1 bound to the PAT acts as a scaffold to recruit ataxin-2 via PABC-PAM2 contact. The C2 region of ataxin-2 triggers the aggregation of PABPC1 in complex with mRNAs, leading to the promotion of SG formation. Consistent with this model, we observed PABPC1 overexpression suppressed SG formation provably through disruption of the ataxin-2–PABPC1–mRNA complex (Supplementary Figure S5).

In this study, we have identified C1 region (925–1079 aa) as the minimum region required for its localization to SGs. However, compared with the larger 906–1095 aa region, excluding the PAM2 showed rather inefficient localization (Figure 5D and Supplementary Table S4). These results suggest some role played by the PAM2 motif in the localization to SGs. A recent study demonstrated that the PAM2 motif acts as a condensation switch for ataxin-2 wherein PABPC1 binding to the PAM2 solubilizes ataxin-2 to prevent spontaneous condensation, allowing mixing of ataxin-2 with SGs under stress condition (41). Deleting the PAM2 prevented the proper mixing of ataxin-2 into granules. The inefficient localization of 925–1095 aa region, which does not contain PAM2 as well as C2 region, might partly show such de-mixing into SGs and 906–1079 might be the region required and sufficient for localization to SGs.

We previously demonstrated that arsenite stress induces global stabilization of the mRNA PAT by the selective degradation of Tob and Pan3 proteins, which are components of the general mRNA deadenylases that bind to PABPC1(40). Both Tob and Pan3 directly bind to PABPC1 to mediate the deadenylation of the mRNAs by recruiting the catalytic subunit of the deadenylases Caf1-Ccr4 and Pan2, respectively, through their PAM2 motifs (22,28,30,33). As arsenite stress selectively induces the proteasome-mediated degradation of Tob and Pan3 (40), the degradation of the PAM2-containing proteins may enable the access of another PAM2-containing protein ataxin-2 to the PABC/MLLE domain of PABPC1 to promote SG formation.

Recently, we have identified ataxin-2 as a cytoplasmic polyadenylation specificity factor that induces post-transcriptional polyadenylation of the target mRNA by recruiting the noncanonical poly(A) polymerase PAPD4 (34). The C-terminal region (906–1095 aa) of ataxin-2 is responsible for inducing the polyadenylation (33). This region contains both the PAM2 motif and the C1 region required for the localization of ataxin-2 to the SGs, and constitutes a region required for promoting SG formation. The coincidence of a region involved in cytoplasmic polyadenylation with a region required for SG formation suggests that ataxin-2-mediated mRNA polyadenylation may also be involved in SG formation. One intriguing possibility is that, in response to stress, ataxin-2 induces posttranscriptional polyadenylation of the target mRNAs to promote localization of the long poly(A)-tailed mRNAs to SGs and to promote SG formation. Another possibility is that ataxin-2 may function to sustain SGs by mediating polyadenylation of stored mRNAs under stress conditions and to prepare for their reuse upon recovery from stress. Because the carboxy-terminal region of ataxin-2 is predicted to be a low-complexity and intrinsically disordered unstructured region (41,42), it is reasonable to assume that the self-aggregation of the C2 region of ataxin-2 contributes to the formation of a liquid droplet that promotes SG formation. Because liquid–liquid phase separation involves a 3D mesh network structure by multivalent interaction, weak interactions such as π–π interactions and cation–π interactions would be required in addition to the self-aggregation of the C2 region. Although we have not examined these possibilities, the polyadenylation reaction may occur efficiently in the membrane-less organelles of SGs.

While SGs require mRNAs with long PATs for their formation, we have also shown that PB formation requires mRNAs with short PATs: (i) global shortening of the mRNA PAT by expressing Pan2 and Caf1 deadenylases promotes PB formation (Supplementary Figure S3), (ii) global lengthening of the PAT by expressing dominant-negative mutants of deadenylases Pan2 D1083A and Caf1 D161A inhibited PB formation (Supplementary Figure S3), which is in sharp contrast to the SG formation. These results are consistent with previous observation that PB formation requires mRNA deadenylases (18). Although we did not further explore the mechanism of PB formation, RNA-binding proteins other than PABPC1 and ataxin-2 are likely involved in the formation of PBs.

## Supplementary Material

gkae497_Supplemental_File

## Data Availability

All data are available in the main text or the supplementary data.

## Notes

Present address: Ryota Yamagishi, Department of Pathophysiology, Osaka Metropolitan University, Graduate School of Medicine, Osaka, Japan.
