# Supplementary material for: Concerted action of ataxin-2 and PABPC1-bound mRNA poly(A) tail in the formation of stress granules
Source: Nucleic Acids Res. 2024 Jun 13;52(15):9193–209. doi: 10.1093/nar/gkae497 (PMC11347130; doi:10.1093/nar/gkae497)
Supplement: gkae497_Supplemental_File [file gkae497_supplemental_file.pdf]

SUPPLEMENTARY DATA

Concerted action of ataxin-2 and PABPC1-bound mRNA poly(A) tail in the formation of stress granules

Ryota Yamagishi[1, 2, †], Hiroto Inagaki[1, †], Jun Suzuki[1], Nao Hosoda[1], Haruka Sugiyama[1], Kazunori Tomita[1], Takashi Hotta[1] and Shin-ichi Hoshino[1,*]

[1] Department of Biological Chemistry, Graduate School of Pharmaceutical Sciences, Nagoya City University, Nagoya 467-8603, Japan.
[2] Current address: Department of Pathophysiology, Osaka Metropolitan University, Graduate School of Medicine, Osaka, Japan.

* To whom correspondence should be addressed. Tel: +81-52-836-3427; Fax: +81-52-836-3427; Email: hoshino@phar.nagoya-cu.ac.jp

† The first two authors should be regarded as Joint First Authors.

This PDF includes: Supplementary Figure S1-S5

SUPPLEMENTARY MATERIALS AND METHODS

Plasmids

To construct pCMV-2×TO-5×Myc and pCMV-2×TO-5×Myc-Pan2, 2×TO fragments prepared by hybridizing HI161 and HI162 primers were inserted into SacI-treated pCMV-5×Myc and pCMV-5×Myc-Pan2, respectively, using NEBuilder HiFi DNA Assembly (NEB). To construct pCMV-5×Flag-BGG, β-globin genomic DNA was PCR-amplified using pFlag-CMV5/TO-BGG as a template and the primer pair NH047/NH048. The resulting fragment was digested with HindIII and inserted into the HindIII and EcoRV sites of pCMV-5×Flag. Primers used in this study are as follows:

NH047: TTTAAGCTTGTGCATCTGACTCCTGAGGAG

NH048: TTTCTGCAGAAGCAAGAGAACTGAGTGGAG

HI161:AGGTCTATATAAGCAGAGCTCTCCCTATCAGTGATAGAGATCTCCCTATCAGTG ATAGAGATCGAGCTCGTTTAGTGAACCGTCAGAT

HI162:ATCTGACGGTTCACTAAACGAGCTCGATCTCTATCACTGATAGGGAGATCTCTA TCACTGATAGGGAGAGCTCTGCTTATATAGACCT

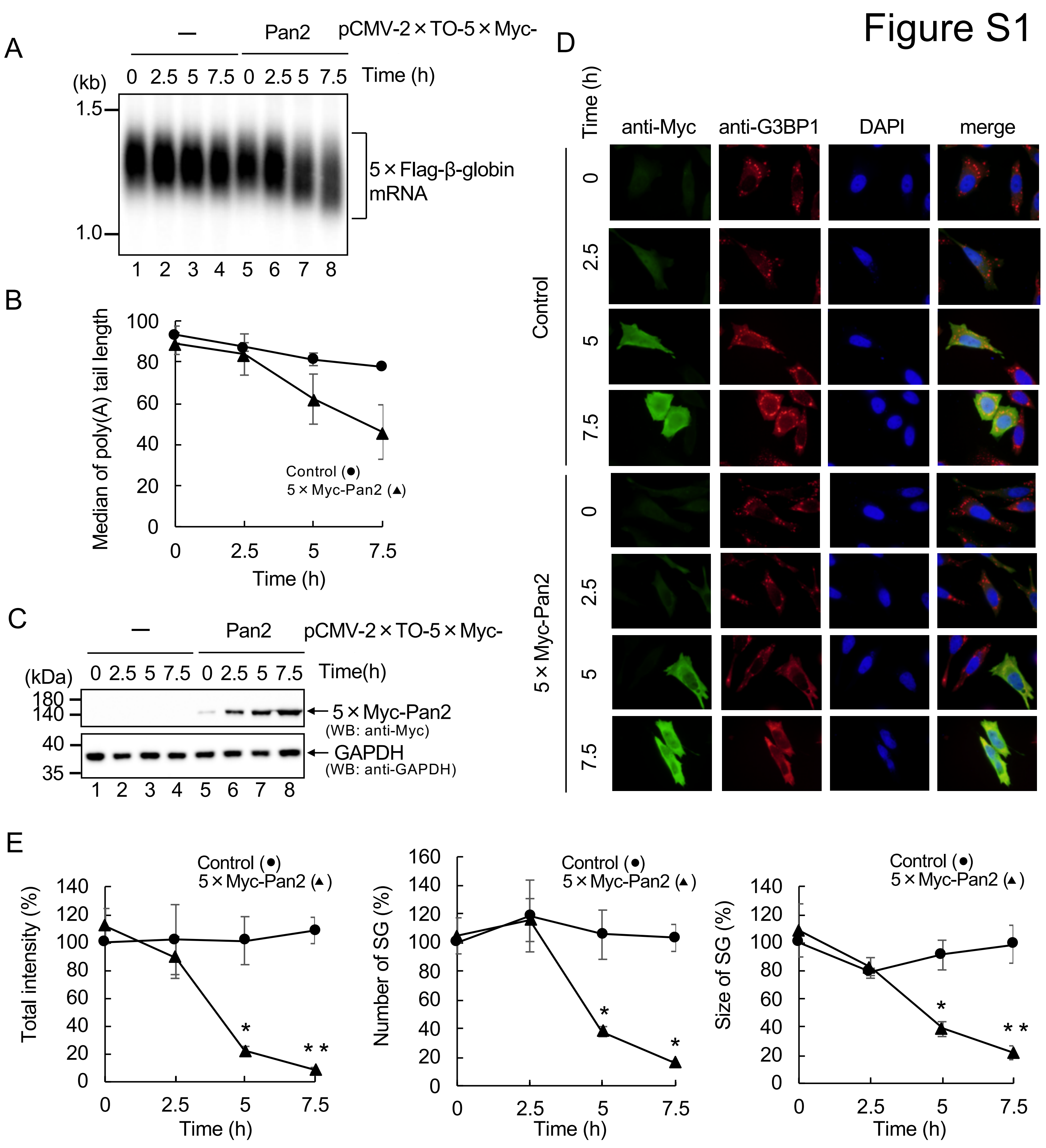

Figure S1
A
— Pan2 pCMV-2×TO-5×Myc-
(kb) 0 2.5 5 7.5 0 2.5 5 7.5 Time (h)
1.5
1.0
5×Flag-β-globin mRNA
1 2 3 4 5 6 7 8
B
Median of poly(A) tail length
Time (h)
Control (●)
5×Myc-Pan2 (▲)
C
— Pan2 pCMV-2×TO-5×Myc-
(kDa) 0 2.5 5 7.5 0 2.5 5 7.5 Time(h)
180
140
40
35
5×Myc-Pan2
(WB: anti-Myc)
GAPDH
(WB: anti-GAPDH)
1 2 3 4 5 6 7 8
D
Time (h)
anti-Myc
anti-G3BP1
DAPI
merge
Control
5×Myc-Pan2
E
Total intensity (%)
Number of SG (%)
Size of SG (%)
Time (h)
Control (●)
5×Myc-Pan2 (▲)

Supplementary Figure S1. Pan2 overexpression induces suppression of SG formation concomitant with the shortening of poly(A) tail.

T-REx-HeLa cells were co-transfected with the pCMV-5×Flag-BGG reporter plasmid and either pCMV-2×TO-5×Myc or pCMV-2×TO-5×Myc-Pan2. One day after the transfection, 5×Myc-Pan2 was induced for 2 h by tetracycline treatment. After the induction, analysis was conducted at the specified time. (A) 5×Flag-β-globin mRNA was detected by northern blot analysis. (B) Median of poly(A) tail length was calculated based on Figure S1A. Results are the average of three independent experiments and shown as means ± SD. (C) Proteins were analyzed by western blotting using the indicated antibodies. (D) HeLa cells were treated with arsenite (0.5 mM) for 30 min and exogenous Pan2 and endogenous G3BP1 were detected by indirect immunofluorescence. (E) For quantitative analysis, the total intensity, size and number of SGs were calculated based on Figure S1D using CellSens software. The quantitative value of SGs in the control cells of 0-h time point was defined as 100%. *, $p < 0.05$; **, $p < 0.01$.

Figure S2

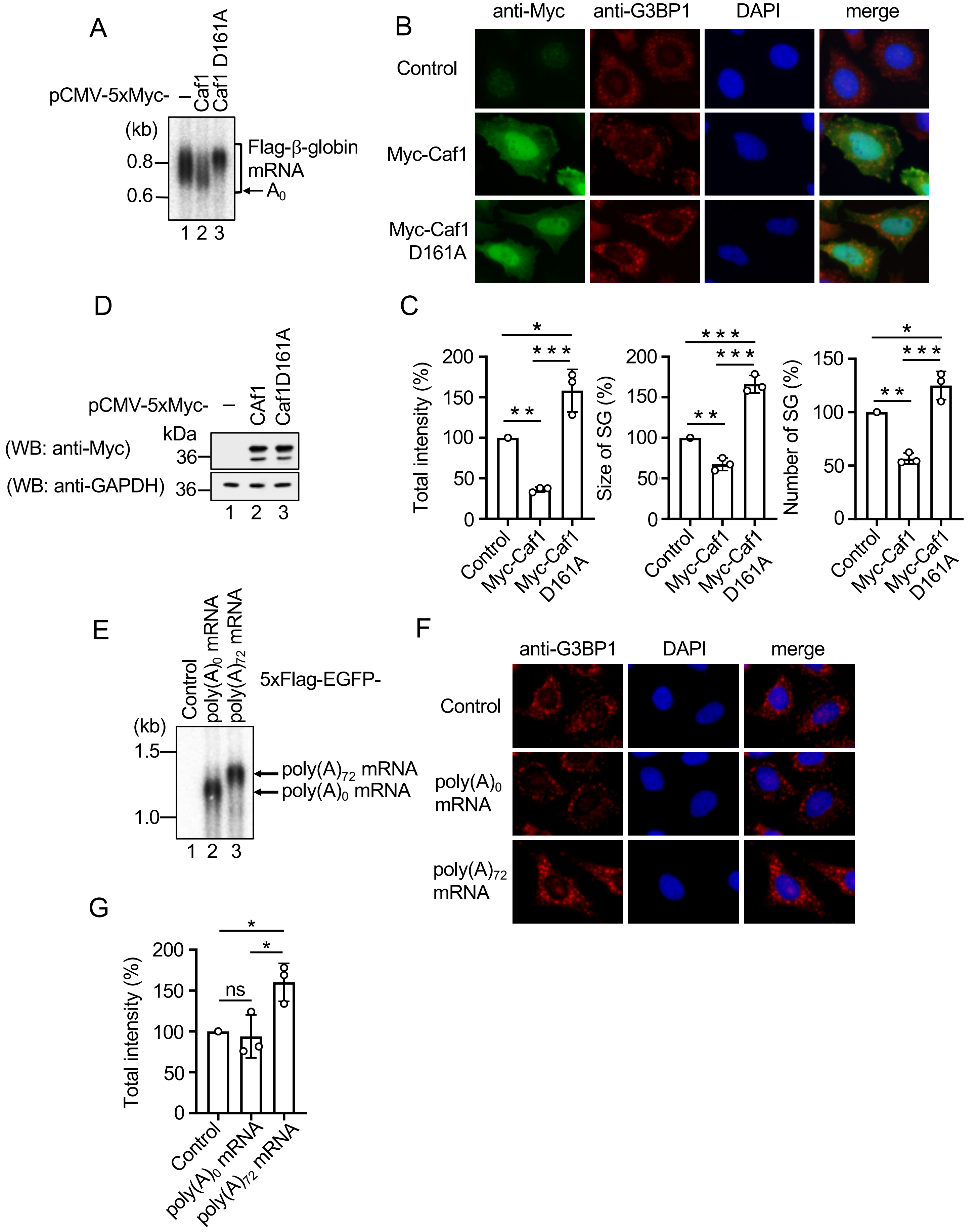

A
pCMV-5xMyc-
–
Caf1
Caf1 D161A
(kb)
0.8
0.6
Flag-β-globin mRNA
A0
1 2 3
B
anti-Myc
anti-G3BP1
DAPI
merge
Control
Myc-Caf1
Myc-Caf1 D161A
D
pCMV-5xMyc-
–
CAf1
Caf1D161A
kDa
(WB: anti-Myc)
36
(WB: anti-GAPDH)
36
1 2 3
C
Total intensity (%)
Size of SG (%)
Number of SG (%)
Control
Myc-Caf1
Myc-Caf1 D161A
E
Control
poly(A)0 mRNA
poly(A)72 mRNA
5xFlag-EGFP-
(kb)
1.5
1.0
poly(A)72 mRNA
poly(A)0 mRNA
1 2 3
F
anti-G3BP1
DAPI
merge
Control
poly(A)0 mRNA
poly(A)72 mRNA
G
Total intensity (%)
ns
Control
poly(A)0 mRNA
poly(A)72 mRNA

Supplementaly Figure S2. Poly(A) tail is required for SG formation.

A to D, HeLa cells were co-transfected with the pFlag-CMV5/TO-BGG reporter plasmid and either pCMV-Myc, pCMV-5×Myc-Caf1 or pCMV-5×Myc-Caf1 D161A. (A) β-globin mRNA was detected by northern blot analysis. (B) HeLa cells were treated with arsenite (0.5 mM) for 30 min, and exogenous Caf1 and endogenous G3BP1 were detected by indirect immunofluorescence. (C) For quantitative analysis, the total intensity, size, and number of SGs were calculated based on Figure S1C using MetaMorph software. The quantitative value of SGs in the control cells was defined as 100%. E to G, HeLa cells were transfected with in vitro-transcribed 5×Flag-EGFP poly$(A)_0$ mRNA or 5×Flag-EGFP poly$(A)_{72}$ mRNA. (D) Proteins were analyzed by western blotting using the indicated antibodies. (E) Transfected mRNAs were detected by northern blot analysis. (F) Cells were treated with arsenite (0.5 mM) for 30 min, and endogenous G3BP1 was detected by indirect immunofluorescence. (G) For quantitative analysis, the total intensity of the SGs was calculated based on Figure S2F using MetaMorph software. The quantitative value of SGs in the control cells was defined as 100%. Results are the average of three independent experiments and shown as means ± SD. *, $p < 0.05$; **, $p < 0.01$; ***, $p < 0.001$.

Figure S3

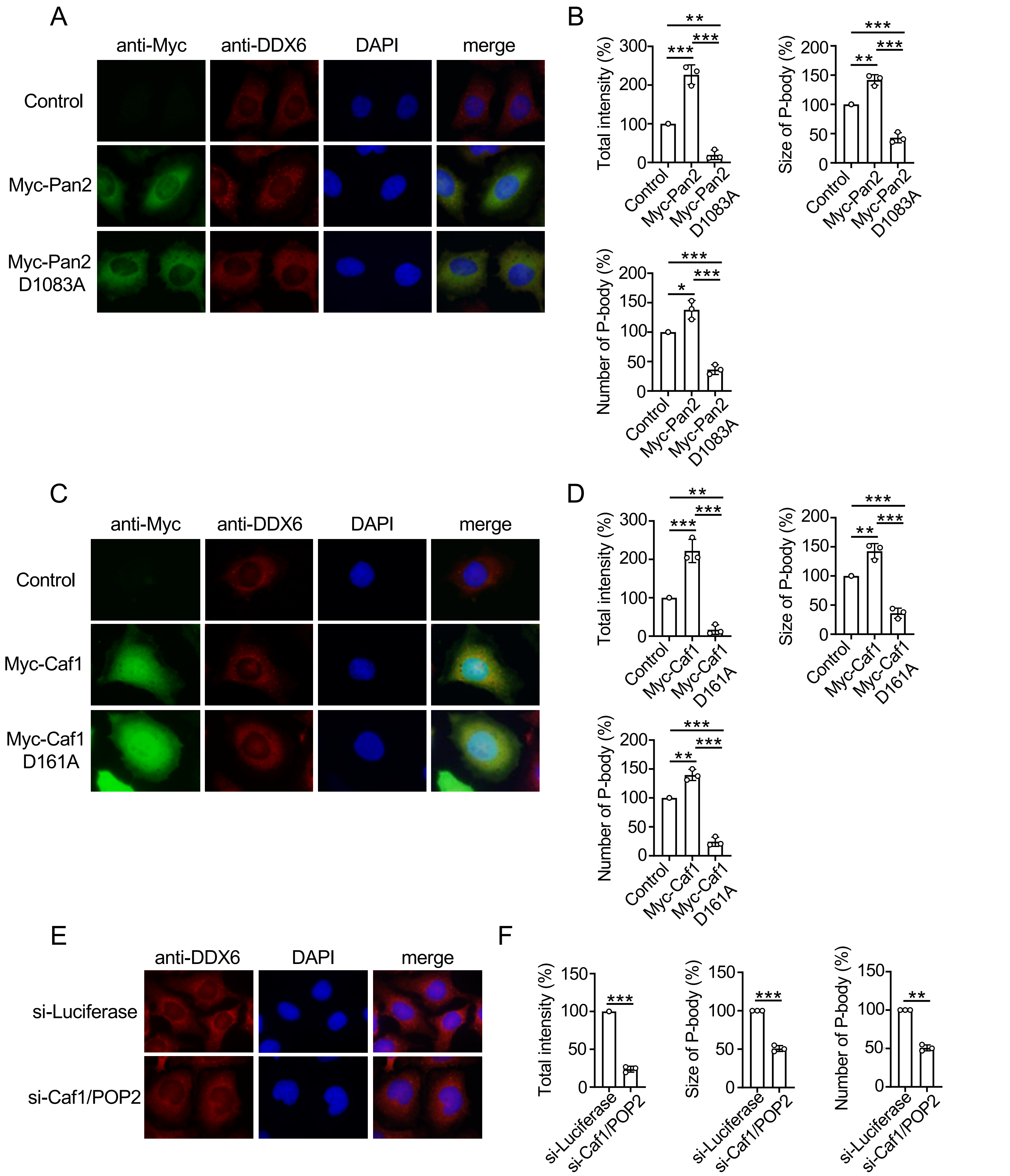
A
anti-Myc
anti-DDX6
DAPI
merge
Control
Myc-Pan2
Myc-Pan2 D1083A
B
Total intensity (%)
Size of P-body (%)
Number of P-body (%)
Control
Myc-Pan2
Myc-Pan2 D1083A
C
anti-Myc
anti-DDX6
DAPI
merge
Control
Myc-Caf1
Myc-Caf1 D161A
D
Total intensity (%)
Size of P-body (%)
Number of P-body (%)
Control
Myc-Caf1
Myc-Caf1 D161A
E
anti-DDX6
DAPI
merge
si-Luciferase
si-Caf1/POP2
F
Total intensity (%)
Size of P-body (%)
Number of P-body (%)
si-Luciferase
si-Caf1/POP2

Supplementaly Figure S3. The effect of mRNA deadenylation on P-body formation was the opposite of that on SG formation.
(A) HeLa cells were transfected with pCMV-Myc, pCMV-5×Myc-Pan2 or pCMV-5×Myc-Pan2 D1083A. Exogenous Pan2 and endogenous DDX6 were detected using indirect immunofluorescence. (B) For quantitative analysis, the total intensity, size, and number of PBs were calculated based on Figure S3A using MetaMorph software. The quantitative value of PBs in the control cells was defined as 100%. (C) HeLa cells were transfected with pCMV-Myc, pCMV-5×Myc-Caf1, or pCMV-5×Myc-Caf1 D161A. Exogenous Caf1 and endogenous DDX6 were detected by indirect immunofluorescence. (D) For quantitative analysis, the total intensity, size, and number of PBs were calculated based on Figure S3C using MetaMorph software. The quantitative value of PBs in the control cells was defined as 100%. (E) HeLa cells were transfected with luciferase siRNA or Caf1/POP2 siRNA. Endogenous DDX6 was detected using indirect immunofluorescence. (F) For quantitative analysis, the total intensity, size, and number of PBs were calculated based on Figure S3E using MetaMorph software. The quantitative value of PBs in luciferase siRNA-transfected cells was defined as 100%. Results are the average of three independent experiments and shown as means ± SD. **, $p < 0.01$; ***, $p < 0.001$.

Figure S4

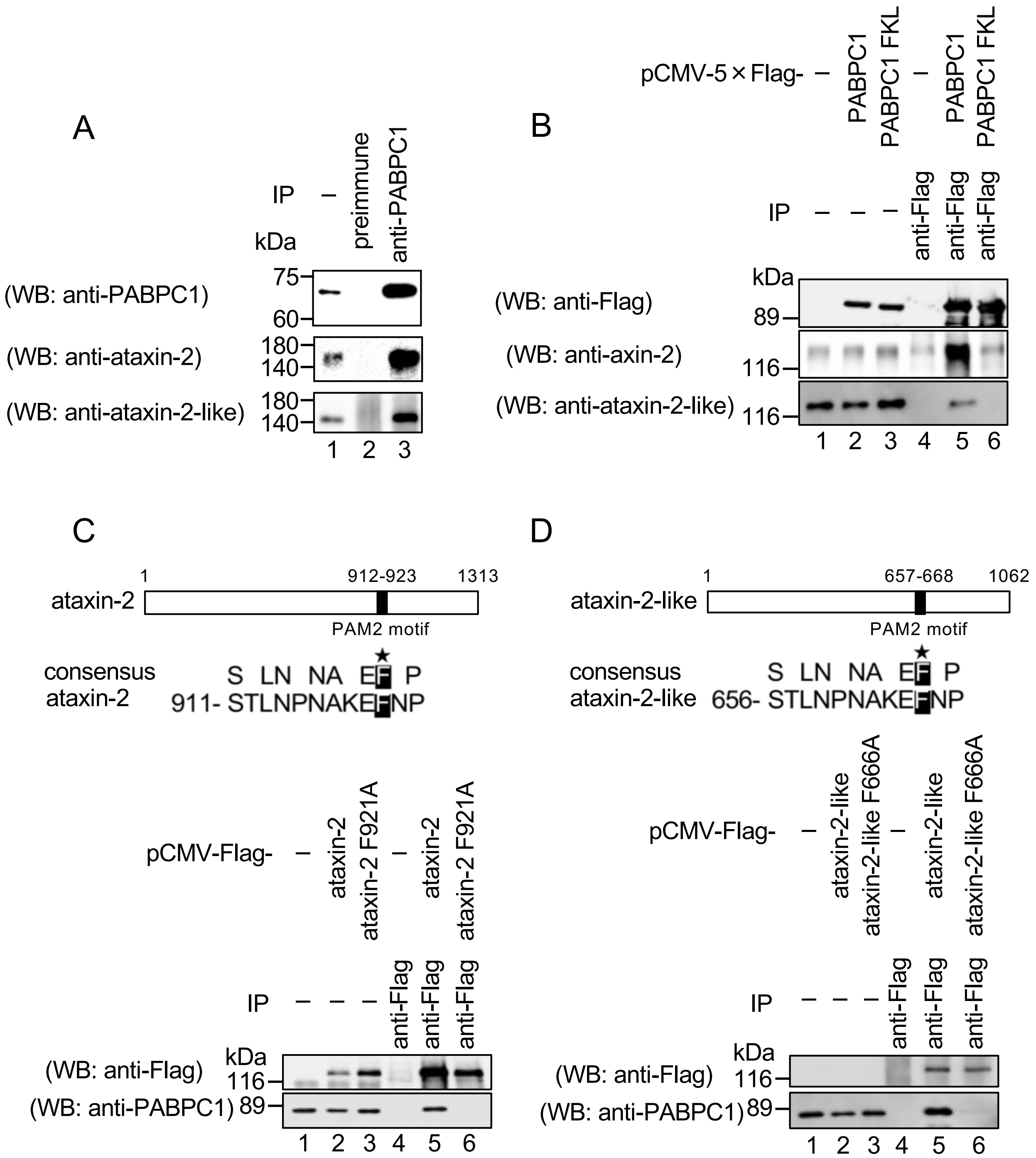

A
IP
–
preimmune
anti-PABPC1
kDa
(WB: anti-PABPC1)
75
60
(WB: anti-ataxin-2)
180
140
(WB: anti-ataxin-2-like)
180
140
1 2 3
B
pCMV-5×Flag-
–
PABPC1
PABPC1 FKL
–
PABPC1
PABPC1 FKL
IP
– – –
anti-Flag
anti-Flag
anti-Flag
kDa
(WB: anti-Flag)
89
(WB: anti-axin-2)
116
(WB: anti-ataxin-2-like)
116
1 2 3 4 5 6
C
1
912-923
1313
ataxin-2
PAM2 motif
★
consensus
S LN NA EF P
ataxin-2
911- STLNPNAKEFNP
pCMV-Flag-
–
ataxin-2
ataxin-2 F921A
–
ataxin-2
ataxin-2 F921A
IP
– – –
anti-Flag
anti-Flag
anti-Flag
kDa
(WB: anti-Flag)
116
(WB: anti-PABPC1)
89
1 2 3 4 5 6
D
1
657-668
1062
ataxin-2-like
PAM2 motif
★
consensus
S LN NA EF P
ataxin-2-like
656- STLNPNAKEFNP
pCMV-Flag-
–
ataxin-2-like
ataxin-2-like F666A
–
ataxin-2-like
ataxin-2-like F666A
IP
– – –
anti-Flag
anti-Flag
anti-Flag
kDa
(WB: anti-Flag)
116
(WB: anti-PABPC1)
89
1 2 3 4 5 6

Supplementaly Figure S4. Ataxin-2 and ataxin-2-like bind to the PABC/MLLE domain of PABPC1 via their PAM2 motifs.

(A) HeLa cell extracts were subjected to immunoprecipitation (IP) using anti-PABP or preimmune serum as non-specific controls. The immunoprecipitation (lanes 2 and 3) and input (lane 1) were analyzed by western blotting using the indicated antibodies. (B) HeLa cells were transfected with pCMV-5×Flag (lanes 1 and 4), pCMV-5×Flag-PABPC1 (lanes 2 and 5), or pCMV-5×Flag-PABPC1-FKL (lanes 3 and 6). The cells extracts were subjected to IP using an anti-Flag antibody. The immunoprecipitation (lanes 4-6) and inputs (lanes 1-3) were analyzed by western blotting using the indicated antibodies. (C) HeLa cells were transfected with pCMV-Flag (lanes 1 and 4), pCMV-Flag-ataxin-2 (lanes 2 and 5), or pCMV-Flag-ataxin-2-F921A (lanes 3 and 6). The cells extracts were subjected to an IP assay and western blotting as described in Figure 6A. (Top) ataxin-2 protein is schematically represented. The black bar indicates the putative PAM2 motif. (D) HeLa cells were transfected with pCMV-Flag (lanes 1 and 4), pCMV-Flag-ataxin-2-like (lanes 2 and 5), or pCMV-Flag-ataxin-2-like-F666A (lanes 3 and 6). The cells extracts were subjected to an IP assay and western blotting, as described in Figure 6A. (Top) ataxin-2-like protein is schematically represented. The black bar indicates the putative PAM2 motif.

Figure S5

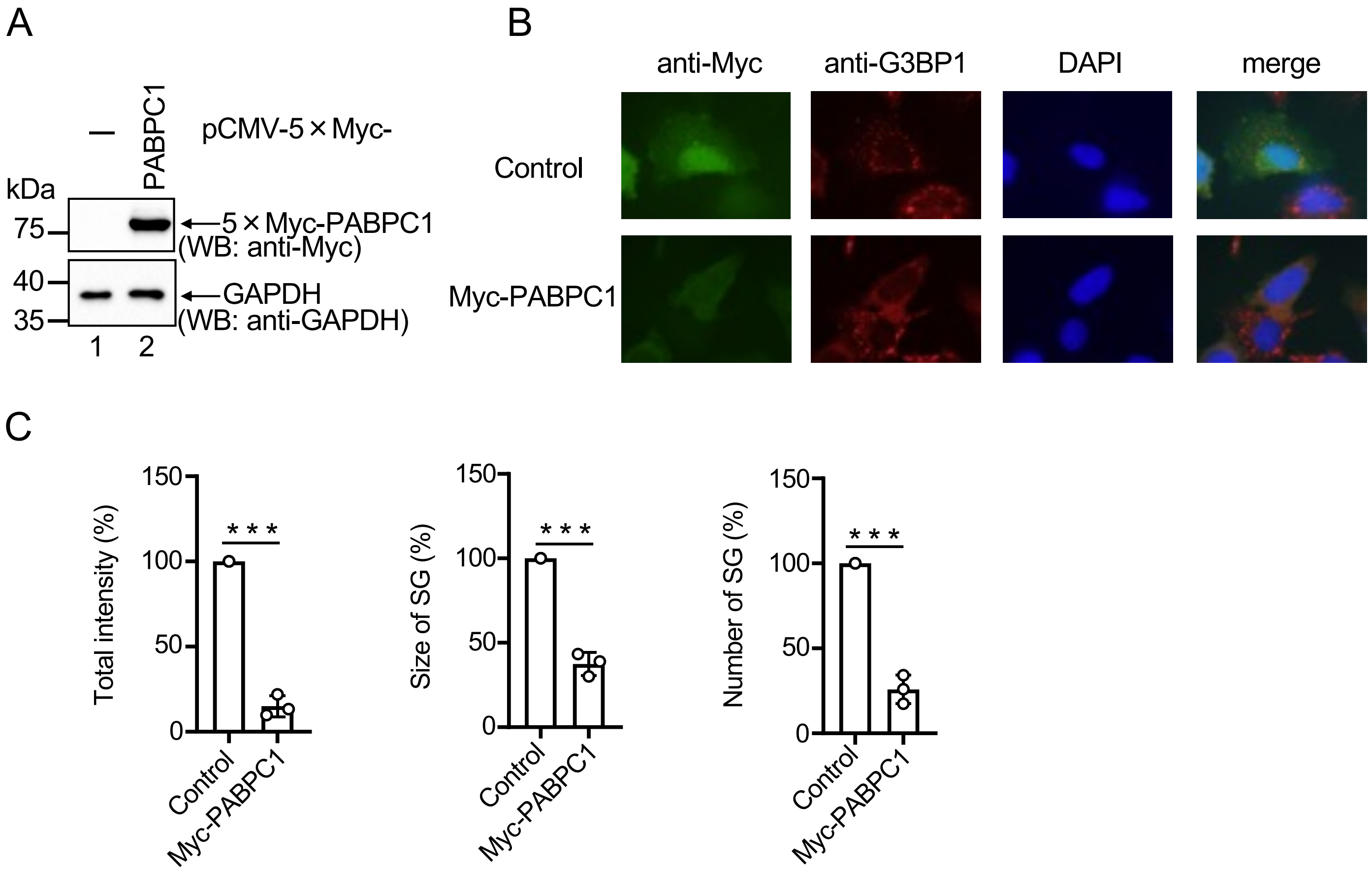

Supplementary Figure S5 . PABPC1 overexpression suppresses SG formation.
HeLa cells were transfected with pCMV-5×Myc, pCMV-5×Myc-PABPC1. (A) Proteins were analyzed by western blotting using the indicated antibodies. (B) HeLa cells were treated with arsenite (0.5 mM) for 30 min, and 5×Myc-PABPC1 and G3BP1 were detected by indirect immunofluorescence. (C) For quantitative analysis, the total intensity, size, and number of SGs were calculated based on (B) using cellSens software. The quantitative value of SGs in the control cells was defined as 100%. ***, $p < 0.001$.

Table S1

Supplementary Table S1. Primers used in this study

name	Sequence
NH751	ATGAACCCCAGTGCCCCCAGCTAC
NH348	CCCTGAGTCGACTTAAACAGTTGGAACACC
NH554	AATATCTTCATTAAAAATCTGGAC
NH555	ACCGACACCGCTTTTGCGAAGTGATGGATCAC
NH116	CGCAAGCTTCGCTCAGCGGCCGCAGCTCC
NH266	GCTACCAAAACAGTCTGAGGC
NH258	TCCCTCCGCCTCAGACTGTTT TGG
NH262	CCTATCATCATTTTCCAG
NH112	CGAAAGCTTAGGATGGTTCATATACTTAC
NH113	AGGGTCGACTTACAACTGCTGTTGGTGGTG
NH134	GCAAAGGAGTTCAACCCACGT
NH135	ATTGGGATTCAATGTTGATTT
NH144	CTTAATTCGGGCCTCCATGGC
NH145	GATCCATGACATGATAAGATACA
ry137	CTTGCTGAGCAAGTTAGGAAATC
ry138	CTAGGGCCTCCCGCCGCCGGAGGT
ry139	CTATCCATTAACTACTCTTTGGT
ry140	CTATGCGTCTTTCTTCTCTTCCT
HT010	TCCTTCTCTCAGCCAAAGCCT
HT012	ACTGAATTCGGGGCTCATGAGCAGACGCAT
HT015	CTCGTCGACCTAGTACTGAGTTGCTGAAGA
HT018	GATGTCGACTTACAACTGCTGTTGGTGGTG
HT020	GACGAATTCGAGCAAGTTAGGAAATCAACA
HT025	GGGCTGATTTGGGAACTGCTG
HT026	TGGTGCCATCATTCTAGCATT
HT027	GAATTCAAGCTTATCCGATTT
HT028	TAAGTCGACATCGATAGATCT
NH219	CTGGAATTCATGTTGAAGCCTCAGCCGC
NH220	GGTGAATTCCCAGGACAATCTTCAGTTC
NH248	GCCAATCCTACAAAGCCTCTG
NH249	CTCCTTAGCATTAGGGTTC
NH733	ACCCTCGACCCCACCATGGCATCAATGGAT
NH732	ATCGAATTCAAGCTTAGTACAGCTCGTCCATGCC
NH734	CTTGAATTCGATATCGTCGACGCTCGCTTTCTTGCTGCTCAATTTCT
NH735	CTTGAATTCGATATCGTCGACGCTCGCTTTCTTGCTGTCCAATTTCT
NH746	AGATCTTTTTTTTTTTTTTTTTTTTTTTTTTTTTTCTAGACATCATTGCAATGAAAA
NH770	GTCTCTTTTTTTTTTTTTTTTTTTTTTTTTTTTTTTTTTTTTTTTTTTTTTTTTTTTTT<br>TTTTTTTTTTTTTTTTTTTCTAGACATCATTGCAATGAAAA
NH773	CTTACACGCTGAACTTGTGGCCGTT

Table S2

Supplementary Table S2. siRNA used in this study

name	Sequence
si-Luciferase	CGUACGCGGAAUACUUCGA dTdT
si-Caf1	CAUCUGGUAUCCAGUUUAA dTdT
si-POP2	GUUGCUGAUCAGUUGGAUU dTdT
si-PABPC1	GUGGAGUAGGCAACAUAUU dTdT
si-ataxin-2	CUUACAGUCCGAAGUGUGA dTdT
si-ataxin-2-like	CUUCAACUAUGCUACUAAA dTdT

Supplementary Table S3

For quantitative analysis, the total intensity of ataxin-2-positive SGs were calculated based on Fig. 5A using cellSens software. The quantitative value of ataxin-2-positive SGs in Flag-ataxin-2 expressed cells was defined as 100%. Results are the average of three independent experiments and shown as means ± SD.

Total intensity(Flag/G3BP1) (%)	
Flag-ataxin-2	100
Flag-ataxin-2(1-240)	0.10±0.17
Flag-ataxin-2(1-548)	0.02±0.04
Flag-ataxin-2(1-905)	1.1±0.4
Flag-ataxin-2(906-1313)	89.0±66.4

Table S4

Supplementary Table S4

For quantitative analysis, the total intensity of ataxin-2-positive SGs were calculated based on Fig. 5D using cellSens software. The quantitative value of ataxin-2-positive SGs in Flag-GST-ataxin-2(906-1313) expressed cells was defined as 100%. Results are the average of three independent experiments and shown as means ± SD.

Total intensity (Flag/G3BP1) (%)	
Flag-GST	0
Flag-GST-ataxin-2(906-1313)	100
Flag-GST-ataxin-2(906-1095)	167.4±75.6
Flag-GST-ataxin-2(1096-1313)	0.29±0.26
Flag-GST-ataxin-2(925-1095)	16.8±6.9
Flag-GST-ataxin-2(925-1079)	16.9±8.3
Flag-GST-ataxin-2(925-1050)	0
